# Highly efficient oxygen evolution reaction via facile bubble transport realized by three-dimensionally stack-printed catalysts

**DOI:** 10.1038/s41467-020-18686-0

**Published:** 2020-10-01

**Authors:** Ye Ji Kim, Ahyoun Lim, Jong Min Kim, Donghoon Lim, Keun Hwa Chae, Eugene N. Cho, Hyeuk Jin Han, Ki Ung Jeon, Moohyun Kim, Gun Ho Lee, Gyu Rac Lee, Hyun S. Ahn, Hyun S. Park, Hyoungsoo Kim, Jin Young Kim, Yeon Sik Jung

**Affiliations:** 1 Department of Materials Science and Engineering, Korea Advanced Institute of Science and Technology (KAIST), Daejeon, 34141 Republic of Korea; 2 Center for Hydrogen & Fuel Cell Research, Korea Institute of Science and Technology (KIST), Seoul, 02792 Republic of Korea; 3 Materials Architecturing Research Center, Korea Institute of Science and Technology (KIST), Seoul, 02792 Republic of Korea; 4 Department of Chemistry, Yonsei University, Seoul, 03722 Republic of Korea; 5 Advanced Analysis Center, Korea Institute of Science and Technology (KIST), Seoul, 02792 Republic of Korea; 6 Department of Mechanical Engineering, Korea Advanced Institute of Science and Technology (KAIST), Daejeon, 34141 Republic of Korea

**Keywords:** Electrocatalysis, Electrocatalysis, Nanoscale materials

## Abstract

Despite highly promising characteristics of three-dimensionally (3D) nanostructured catalysts for the oxygen evolution reaction (OER) in polymer electrolyte membrane water electrolyzers (PEMWEs), universal design rules for maximizing their performance have not been explored. Here we show that woodpile (WP)-structured Ir, consisting of 3D-printed, highly-ordered Ir nanowire building blocks, improve OER mass activity markedly. The WP structure secures the electrochemically active surface area (ECSA) through enhanced utilization efficiency of the extended surface area of 3D WP catalysts. Moreover, systematic control of the 3D geometry combined with theoretical calculations and various electrochemical analyses reveals that facile transport of evolved O_2_ gas bubbles is an important contributor to the improved ECSA-specific activity. The 3D nanostructuring-based improvement of ECSA and ECSA-specific activity enables our well-controlled geometry to afford a 30-fold higher mass activity of the OER catalyst when used in a single-cell PEMWE than conventional nanoparticle-based catalysts.

## Abstract (web summary)

Improved design of three-dimensionally nanostructured catalysts for oxygen evolution reaction (OER) can play a key role in maximizing the catalytic performance. Here, the authors show that woodpile-structured iridium consisting of 3D-printed, highly-ordered nanowire building blocks significantly improve OER mass activity.

## Introduction

Water electrolysis is a promising method to produce H_2_, a clean energy source, potentially serving as a central technology in the H_2_-based renewable energy cycle^1^. In particular, polymer electrolyte membrane water electrolyzers (PEMWEs) are attracting strong interest due to their compactness, superior energy efficiency, excellent purity of produced hydrogen, and high production rate^2,3^. However, one of the significant limitations for large-scale industrialization of PEMWEs is that high-cost noble metal catalysts are critical components for facilitating the extremely sluggish kinetics of the oxygen evolution reaction (OER) in an acidic environment^4^.

Among various candidates, Ir-based oxides have been the most widely investigated as a potential catalyst material with high catalytic activity and moderate stability^5,6^. The generation of practical-level current density (>1–2 A cm^−2^) typically requires a high loading (0.5–3 mg cm^−2^) of Ir with a cost that is almost twice that of Pt^7–9^. Therefore, it is highly desirable to develop an innovative technology to raise the mass activity of Ir-based OER catalysts to the targeted level. The mass activity of electrochemical OER catalysts, where the current density normalized by the loaded amount of Ir, is determined by the electrochemically active surface area per loaded mass (ECSA) and ECSA-specific activity (mass activity per ECSA), both of which can be improved by engineering their nanostructures^10–14^.

In particular, the formation of OER catalyst electrodes employing one-dimensional (1D) nanomaterials can be substantially advantageous. Long-range interconnectivity and low-tortuosity pore structures obtained by 1D nanostructures are expected to contribute to the enhancement of ECSA-specific activity by offering high electrical conductivity^15^ and highly efficient transport of evolved gas^12–14^. This is because sufficiently long 1D metallic nanostructures can significantly reduce the number of electrically insulating junction points among catalyst particles^15^, and also can be designed to be beneficial for gas delivery due to the existence of more efficient gas-transporting channels compared to random aggregates of nanoparticles. In previous research, random or vertically aligned nanowires (NWs) have been studied^16–18^, but to date the inter-catalyst space, where gas bubbles can be transmitted, has not been systematically engineered. Gas bubble movement within well-defined transport pathway should be secured to reach a new level of OER electrocatalyst performance, as shown with the enhanced OER activity achieved by facile bubble detachment via transition from the Cassie state to the Wenzel state wetting of bubbles on 2-dimensionally patterned catalytic surfaces^19–21^. Aside from the bubble detachment, bubble coalescence before escaping the catalyst layer should be managed for control over bubble movement in nanostructured electrocatalysts, which was unattainable in previous studies without controllability over long-range 3D geometry.

For augmenting the ECSA of the OER catalysts, maximization of the two-phase contact area between water and catalyst is an essential condition for more effective interaction between the Ir surface, which serves as both catalytic active sites and pathways for the electrons, and an electrolyte^22^. For this purpose, inter-catalyst space should secure the maximized utilization efficiency of the surface area, which is not easy to accomplish with Ir black nanoparticles (one of the state-of-the-art OER catalysts). Furthermore, ionomers, which are typically mixed with Ir nanoparticles for enhancing ionic conductivity in OER electrodes, may reduce the direct contact between the catalytic surface and reactants and also interfere with efficient transport of the generated gas^23^.

Taking all of these factors into account, to maximize catalytic performance, we hypothesize that an “ideal OER electrode” should simultaneously provide (1) efficient utilization of a large surface area per loading amount of catalytic material, (2) well-defined pore structures for highly efficient transport of evolved gas species, (3) long-range connectivity of building blocks for high electronic conductivity, and (4) capability of ionomer-free operation. Although these requirements can be partially satisfied by the above-mentioned catalyst nanostructures, where the water electrolyte can contribute to ionic conductance over a short distance even in the absence of ionomers^24^, it is difficult to find a prior art that demonstrates extensive 3D geometric controllability to deduce practical and universal design rules for high-performance OER catalysts.

Here, we report that 3D-geometry-controlled Ir catalysts with a woodpile (WP) structure suggest a new direction for the OER electrocatalysts to improve the PEMWE performance. The WP structure is attained by systematic engineering of NW building blocks and 3D geometry, which is carried out via ultrahigh-resolution nanotransfer printing. The extensive controllability of the fabrication method enabled control of the NW-to-NW spacing and layer-to-layer alignment angles in the WP nanostructure. With this capability, we reveal that the simultaneous improvements of ECSA and ECSA-specific activity result from uniform access of water reactants to the catalytic surface and easy transport and removal of evolved oxygen gas products through the well-defined pore channels, which is supported by the extensive control of 3D geometry and the scanning electrochemical microscopy (SECM) analysis data combined with microfluidic calculations. Without any alteration of crystallographic structures or oxidation states, WP-structured Ir catalysts achieve a high mass activity value of 140 A/mg (at 1.8 V) in a single-cell PEMWE, which is 30 times higher than that of the state-of-the-art commercial Ir nanoparticle catalysts (Ir black).

## Results

### Fabrication and characterization of WP-structured Ir catalysts

**Fig. 1:**
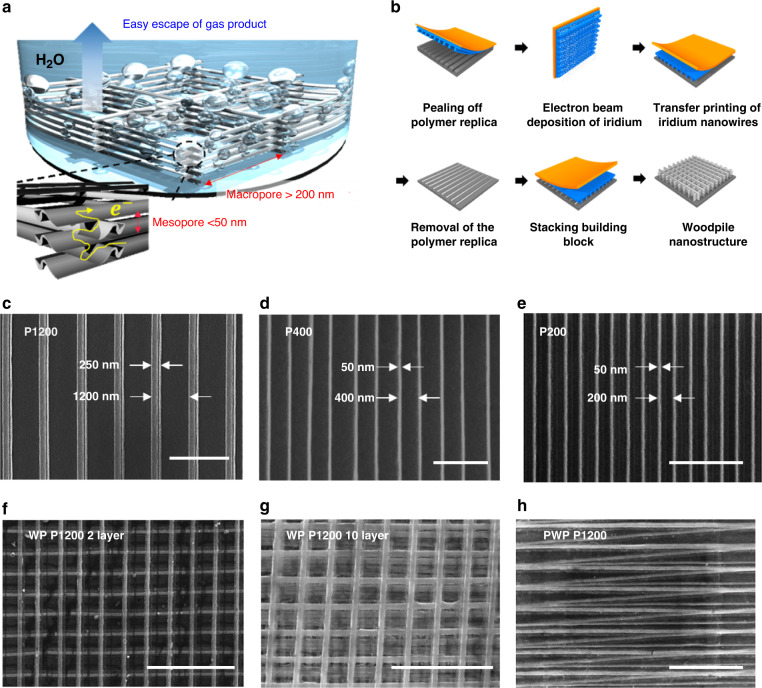
Fabrication of 3D Woodpile-structured Ir catalysts. **a** Illustration of woodpile-structured Ir thin film containing mesopores and macropores for OER. **b** Fabrication process of woodpile-structured Ir with repeating solvent-assisted nanotransfer printing (S-nTP). Building blocks of woodpile structure named according to the periodicity of the nanowire arrays having different line width and period: scale bar in (**c**) is 2 μm, and scale bars in (**d**) and (**e**) are 1 μm. The loaded amount of Ir was controlled quantitatively along the number of stacking layers. **f** 2- and **g** 10-layer perpendicular-stacking of the P1200 building block. **h** Parallel stacking of P1200 building block. Scale bars in (**f**–**h**) indicates 5 μm.

We fabricated 3D WP-structured Ir electrocatalysts (Fig. 1a) with stacking 1D NW arrays processed by solvent-assisted nanotransfer printing (S-nTP), as illustrated in Fig. 1b.

More detailed principles of S-nTP are described in our previous paper^25^. With the S-nTP process, highly ordered NW arrays can be printed on a macroscopic area with controlled size and orientation of NWs. WP-structured Ir was designed to contain well-defined macropores (>200 nm) at the inter-wire space with linkage to the inter-layer mesopores (<50 nm). Figure 1c–e presents the basic building blocks (P1200, P400, and P200; unit of the numbers = nm) of Ir NWs labeled after the periodicity of the NW arrays. Furthermore, the 3D geometry can be tuned using the same building blocks. For example, NWs having a WP structure can be stacked perpendicularly into two layers (Fig. 1f) and ten layers (Fig. 1g, Supplementary Fig. 2a, b), or a parallel woodpile (PWP) structure (Fig. 1h, Supplementary Fig. 2c, d). The extensive controllability in the structure of highly ordered NW array stacks was expected to contribute to clarification of the key factors in enhancing the mass activity of nanostructured catalysts.

**Fig. 2:**
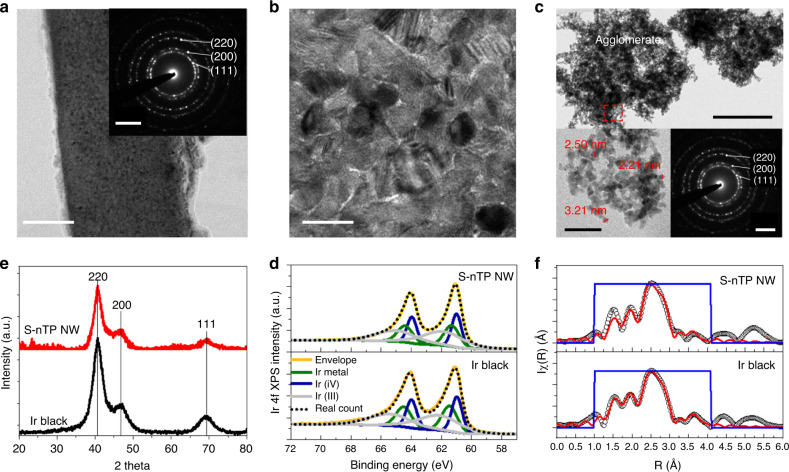
Characteristics of building blocks of Ir woodpile structure. **a** TEM image (inset: corresponding SAED pattern with 5 1/nm scale bar) and **b** HRTEM image of Ir NWs fabricated by S-nTP. Scale bars in (**a**) and (**b**) are 100 and 10 nm, respectively. **c** TEM image (200 nm scale bar), HRTEM image (20 nm scale bar), and SAED patterns (5 1/nm scale bar) of Ir black nanoparticles. **d** Comparison of the XRD patterns of printed NWs and Ir black nanoparticles, showing the same face-centered cubic (FCC) crystal structure. **e** XPS and **f** non-phase corrected Fourier transform of *k*^2^-weight EXAFS data, demonstrating similar oxidation states of Ir in both samples.

In order to investigate whether there is any critical change in the fundamental properties of Ir caused by the S-nTP process, we compared the physiochemical characteristics of 3D WP catalysts with those of Ir black nanoparticles (Premetek)—a commercial Ir catalyst for OER. As the physiochemical characterization in Supplementary Figs. 3 and 4 showed negligible differences among the three types of catalysts built with the building blocks of P1200, P400, and P200, we used the term—S-nTP NWs for indicating all the Ir NWs fabricated with S-nTP method. First, broad X-ray diffraction (XRD) peaks were observed for both the S-nTP Ir NWs and nanoparticle-type Ir black samples (Fig. 2d and Supplementary Fig. 3), which is in line with the selected-area electron diffraction (SAED) patterns (insets of Fig. 2a, c). The diffraction patterns of the samples reveal that both samples are mainly composed of polycrystalline Ir metals having a face-centered cubic (FCC) crystal structure. The peak broadening for the S-nTP NWs and the Ir black can be explained by the small grain size (~3–5 nm in diameter, Fig. 2b) in the NW building blocks and the small particle size (3.4 nm in diameter, Fig. 2c) of the Ir black catalysts.

The chemical nature of the surface species of the as-prepared Ir samples was also examined by X-ray photoelectron spectroscopy (XPS). The Ir 4*f* XPS peaks shown in Fig. 2e, which can be deconvoluted into Ir 4*f*_7/2_ and Ir 4*f*_5/2_ binding peaks^26,27^, indicate there is no noticeable difference between Ir black and S-nTP NWs. In both cases, the two types of iridium oxide peaks comprise ~32 and 36% of the overall Ir 4*f* peak, and the metallic Ir appearing at 60.9 eV^28^ occupies 32% (Supplementary Table 3). Similarly, the O 1*s* XPS spectra (Supplementary Fig. 5) and the ratio of IrO peak to IrOH peak (Supplementary Table 2) were analogous for both samples.

For a more in-depth evaluation of the oxidation state and the local structural environment of Ir, the WP samples composed of S-nTP NWs and nanoparticle-type Ir black reference were further investigated by X-ray absorption near edge structure (XANES) spectroscopy and extended X-ray absorption fine structure (EXAFS) spectroscopy for the Ir-L_III_ edge. The Ir-L_III_ absorption edge, which probes 2*d* to 5*d* electronic transitions, is sensitive to changes in the oxidation state of Ir^29,30^. The XANES spectra (Supplementary Fig. 6) showed no shift of the absorption edge energy among the samples, indicating there is no difference in the average oxidation state of iridium oxide on the surface of the catalysts. For characterizing the local coordination environment of Ir, a two-cluster model was employed to fit the EXAFS data (Fig. 2i and Supplementary Table 4), revealing elongation of the Ir–O bonding distance due to Ir oxide formation on the surface for both Ir black and S-nTP NWs. As the few-atom layer of the Ir metallic surface partly becomes iridium oxide in the air, the formation of iridium hydroxo species (^*^OH) on the surface can elongate the average Ir–O bonding in the sampels^31^. As a result, XPS and XAS analysis data suggest the existence of Ir oxide on the metallic Ir cores, which were identified from the XRD and transmission electron microscopy (TEM) analyses, confirming that the WP samples do not have a prominent difference in oxidation state compared to the Ir black particles.

### Half-cell OER characterization

**Fig. 3:**
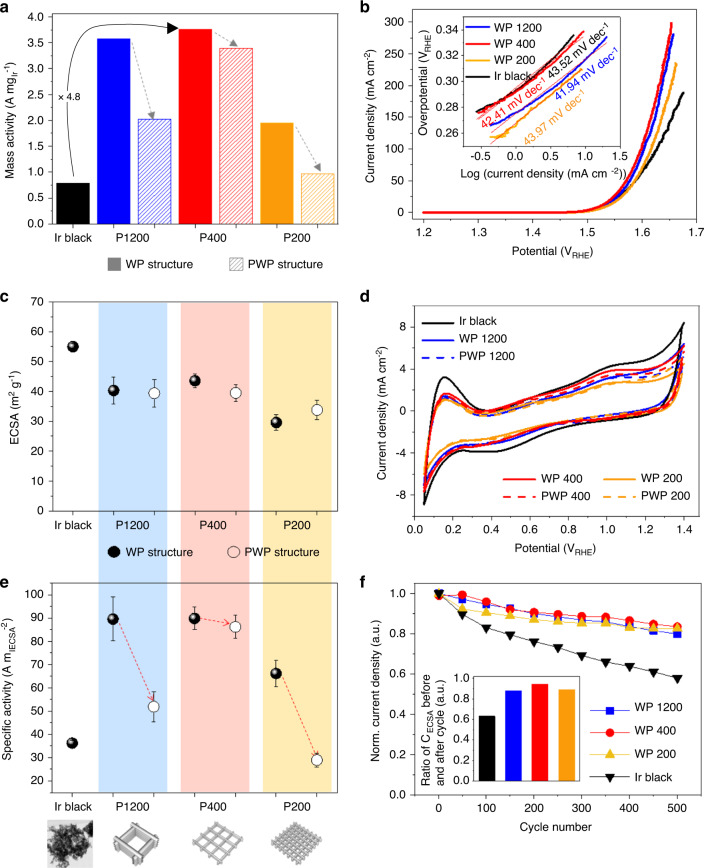
Comparison of OER catalytic half-cell performance. **a** The mass activity of 4-layered 3D WP Ir and Ir black samples, showing a 4.8 times higher mass activity of the WP400 catalyst compared to Ir black. Parallel stacking of NWs resulted in a substantial decrease of mass activity. **b** Linear sweep voltammetry of 4-layered WP Ir and Ir black. The inset presents the Tafel plots for the Ir catalysts, showing analogous Tafel slopes. **c** The ECSA evaluated by the HUPD peak in (**d**) the cyclic voltammetry range of 0.05–1.4 V_RHE_, which oxidizes Ir surfaces electrochemically. **e** ECSA-specific activity calculated by dividing the mass activity by the ECSA. The error bars in (**c**) and (**e**) indicates the standard deviation of measurements. **f** Normalized current density for 500 cycles of repeated chronoamperometry for OER stability test. The inset shows the ratio of integrated surface charge values before and after 500 cycles, presenting a similar trend as the current density.

To elucidate the effect of 3D nanostructuring on the OER performance, we measured the ECSA (ECSA per loaded mass of Ir) of the samples. Solely at the first cycle (Fig. 3d) of the cyclic voltammetry (CV) in Supplementary Fig. 7, it was possible to calculate the ECSA from the hydrogen underpotential deposition (HUPD) peak.

The ECSAs of the three types of 4-layered Ir WP nanostructures (shown in Fig. 3c) were compared to the calculated specific surface area (CSA), which was calculated on the basis of the cross-sectional image of the NWs presented in Supplementary Fig. 1. The Ir utilization efficiency (94–98%, ratio of measured ECSA to CSA, Supplementary Table 1) of the samples fabricated by S-nTP was substantially higher than the utilization efficiency of the Ir black sample (47.7%, Supplementary Table 5). Whereas the CSA of Ir black was at least 160% larger than that of WP samples, the ECSAs of the two types of samples were comparable. The ECSA of the Ir black sample was measured to be at least 26% larger than those of the WP samples, where the ECSAs varied depending on their geometries.

The mass activities of the samples, as shown in Supplementary Table 7, were estimated with the current densities at 1.55 V in the linear sweep voltammetry (LSV) curves (measured at 5 m V s^−1^ scan rate with iR compensation, Fig. 3b and Supplementary Fig. 11a) and their normalization based on the Ir loaded amount in each sample. The highest mass activity among the 3D WP-structured samples was obtained for the WP400 with values of $0.513\,{\mathrm{A}}\,{\mathrm{mg}}_{@1.5\,{\mathrm{V}}_{{\mathrm{RHE}}}}^{ - 1}$, $1.92{\mathrm{A}}\,{\mathrm{mg}}_{@1.53\,{\mathrm{V}}_{{\mathrm{RHE}}}}^{ - 1}$, and $3.76{\mathrm{A}}\,{\mathrm{mg}}_{@1.55\,{\mathrm{V}}_{{\mathrm{RHE}}}}^{ - 1}$. These correspond to 4.8 times the mass activity of Ir black $-0.79\,{\mathrm{A}}\,{\mathrm{mg}}_{@1.55\,{\mathrm{V}}_{{\mathrm{RHE}}}}^{ - 1}$(Fig. 3a) and are comparable to the values $0.38\,{\mathrm{A}}\,{\mathrm{mg}}_{@1.53\,{\mathrm{V}}_{{\mathrm{RHE}}}}^{ - 1}$^13^, and ${\mathrm{0}}{\mathrm{.7}}\,{\mathrm{A}}\,{\mathrm{mg}}_{@1.5\,{\mathrm{V}}_{{\mathrm{RHE}}}}^{ - 1}$^12^ from recent studies, while these studies did not demonstrate device applications for water electrolysis. In terms of reducing overpotential, although some studies demonstrated a low overpotential of 248 mV for the generation of 10 mA cm^−2^ ^32,33^, which is 8.2% smaller than that (270 mV overpotential) of this study, our WP catalyst achieved 187 times higher mass activity at 1.55 V_RHE_.

Consideration of the ECSA (Fig. 3c) of the 3D WP catalyst comparable to the Ir black nanoparticles suggests that the ECSA alone does not explain the 4.8-fold higher mass activity, and thus an additional factor should be taken into account. To elucidate this phenomenon, the ECSA-specific activity (${\mathrm{A}}\,{\mathrm{m}}_{{\mathrm{ECSA}}}^{ - 2}$) should be taken into account (see Fig. 3e and Supplementary Fig. 12). The ECSA-specific activity was obtained by dividing the mass activity (at *E* = 1.55 V_RHE_) by the ECSA. In Fig. 3e and Supplementary Fig. 12, all three types of WP-structured samples show superior ECSA-specific activity compared to the nanoparticle-type Ir black sample, which will be discussed in detail in the next section.

### Effect of 3D geometry on ECSA-specific activity

We attempted to elucidate the effect of 3D nanostructuring on the ECSA-specific activity. As shown in Fig. 3e, the ECSA-specific activity of 3D WP P1200, P400, and P200 samples was 6.2-, 6.0-, and 4.6-fold higher than that of Ir black at 1.55 V_RHE_, respectively. As mentioned above, we confirmed there is no difference in the physiochemical characteristics related to the catalytic activity determined from XPS, XRD, XAS, and TEM analyses and corresponding Tafel slopes (Fig. 3b).

**Fig. 4:**
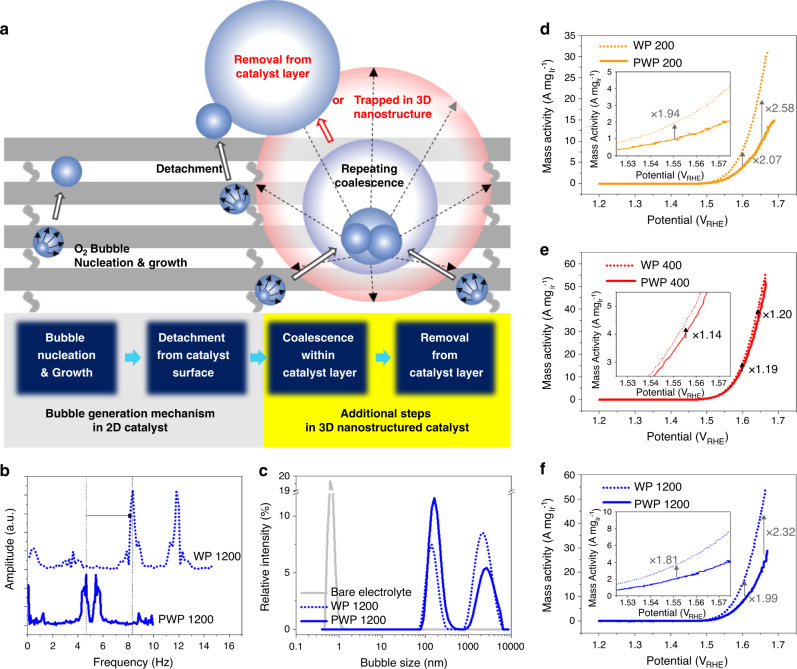
Facile transport of evolved O_2_ gas in the WP catalyst layer. **a** Schematic illustration of bubble generation and removal mechanism within the 3D-nanostructured electrocatalyst. **b** FFT amplitude spectra of currents recorded at the SECM tip (potential of the sample: 1.4 V_Ag/AgC1_). Note that the higher frequency of the peak indicates a faster cycling of formation and detachment of O_2_ bubbles. **c** Dynamic light scattering analysis spectra, showing the size distribution of O_2_ bubbles generated by the WP and PWP catalysts, respectively. Comparison of mass activity depending on the stacking direction (perpendicular vs. parallel) of **d** P200, **e** P400, and **f** P1200. Insets in (**d, e**) shows the narrower range of *x*- and *y*- axis than the entire plots for expansion for expanded view of the comparison of mass activity near 1.55 V_RHE_.

A possible explanation for the superior ECSA-specific activity in the 3D WP-structured catalysts is efficient removal of oxygen bubbles from the catalyst layer. The more than twofold improvement of the mass activity compared to PWP structures (Fig. 3a) despite the use of identical NW building blocks suggests that the open and ordered characteristics of the 3D WP structures can lead to accelerated transport and removal of gas bubbles from the catalyst layer. As illustrated in Fig. 4a, for general OER catalysts including commercial random nanoparticles and our 3D-nanostructured catalysts, it is expected that there are more factors at work beyond simple bubble detachment from catalytic surfaces: bubble coalescence and removal from the catalyst layer. In order to elucidate this mechanism, two hypotheses were set based on the electrochemical analysis data: (A) the open and ordered 3D structure shortens the residence time of bubbles in the internal space of catalyst layer, and (B) longer residence time results in more severe coalescence of bubbles in the fluid.

The first hypothesis (A) was investigated experimentally with SECM (SECM analysis data, Fig. 4b and Supplementary Fig. 13) and theoretically with microfluidic calculations. We conducted SECM characterizations to compare WP P1200, having the highest specific activity among the samples, with PWP 1200. During the SECM measurement, current fluctuations (Supplementary Fig. 13b, c) were monitored by positioning a microelectrode in the proximity of the sample surface, which was biased electrically to evolve gas bubbles. At the narrow space, the formation of insulating gas bubbles leads to a decrease in the current density. As the electrical bias continues, the detachment of the insulating gas bubbles increases the current density again, appearing as a current peak^34^. Extraction of the characteristic frequency of the peaks in current is enabled by fast Fourier transformation (FFT, Fig. 4b), wherein the multiple peaks could be caused by two bubbles evolved from a catalytic site or another activated site at a typical potential range^34^. The higher frequency of the WP nanostructure (8.65 and 11.8 Hz) implies faster formation and removal of O_2_ bubbles during the OER, compared to the PWP nanostructure (4.35 and 5.48 Hz).

Using microfluidic calculations, it is possible to estimate the ratios of theoretical frequencies of bubble removal based on the buoyancy effect (driving force for bubbles to escape from the catalyst layer) and the bubble-catalyst interface surface tension (wetting condition). If the evolved gas bubbles are much smaller than the pore size, $V^{1/3} \ll p_g$, we can treat the bubbles and their transport as simple particles transporting through a porous media, using Darcy’s law^35^. Here, *V* is the volume of gas and *p*_*g*_ is the pore size of the porous structure. We can then conclude that the open and ordered WP1200 structure has almost a five-times larger mass transport rate than the PWP 1200 structure (see Supplementary Note 1). On the other hand, if evolved gas bubbles have similar or larger size than that of the pores, we should consider the force balance between the buoyancy effect (*F*_*b*_) and the bubble-catalyst interface surface tension force (*F*_*s*_), *F*_*b*_ (=Δ*ρVg*) ~ *F*_*s*_ $( = \gamma \ell )$, where Δ*ρ* is the density difference between gas and liquid, *g* is the gravitational acceleration, *γ* is the surface tension, and $\ell$ is the perimeter length for the gas–liquid wetting. We compare a critical volume to be detached from the different nanostructures. It is known that the bubbles’ surface adhesion force can vary depending on the wire alignment; for instance the surface adhesion force of the parallel NWs is much larger than that of the crossing NWs^36,37^. Thus, the critical volume for the PWP geometry is always larger than the WP structure case, i.e., $V_{WP}\left( { = \frac{{8p_g\gamma }}{{{\Delta}\rho g}}} \right)$ < $V_{PWP}\left( { = \frac{{17p_g\gamma }}{{{\Delta}\rho g}}} \right)$ (see the Supplementary Information). The frequency of bubble removal from the catalyst layer (*f*_*b*_) can be scaled from the inertia capillary time scale $\tau _{cap}\sim f_b^{ - 1}\sim \sqrt {{\Delta}\rho V/\gamma }$. Thus, the frequency ratio of bubble removal can be $\frac{{f_{b,WP}}}{{f_{b,PWP}}} = \sqrt {\frac{{17}}{8}} \approx 1.5$, where the volume was considered from the critical volume for each structure (see details in Supplementary Note 2).

The second theoretical model calculated under the assumption of bubble size similar to or larger than pore channel size in the catalyst is consistent with the results of the SECM measurement data. This implies that the bubbles having a size similar to or larger than that of the pores determines the frequency of bubble formation and removal from the catalyst layer. We characterized and confirmed the larger bubbles that must be formed by the coalescence within the 3D catalyst layer via a dynamic light scattering (DLS, Fig. 4c) analysis on the electrolyte, where we conducted LSV once with different 3D geometries (WP 1200 and PWP1200). The larger bubble size (2838 nm) from the PWP1200 structure was estimated to be 19% larger than that (2381 nm) of the bubbles from the more open and ordered WP1200 samples. These results lead us to conclude that the bigger bubbles from PWP tend to have a larger size because of the longer residence time in the catalyst layer, which confirms the second hypothesis (B).

In addition, in the DLS spectra, it can be seen that relative intensity of the bigger bubbles from the PWP structure is relatively lower than that of the smaller bubbles (~150 nm diameter), while the tendency is reversed in the case of bubbles from the WP structure. This can be attributed to the more facile escape of the larger bubbles in the WP structure before growing into much larger ones, which is consistent with the results of the SECM analysis and theoretical calculations. These results suggest that coalescence of bubbles, which is driven by the reduction of the overall surface energy, should be prevented for higher OER activity. As the coalesced gas bubbles within the catalyst layer hinder the approach of the reactant water to the catalytic surface, engineering 3D nanostructures can accelerate the rate of OER per unit ECSA by facilitating the removal of the generated gas bubbles.

There are further experimental results supporting that the ECSA-specific activities of the 3D WP catalysts are governed by the eases of gas bubble transport, which is controlled by the geometric parameters of the 3D catalysts. When the stacking direction of three types of NW building block arrays were changed from perpendicular (WP) to parallel (PWP), the impact of decrease in the ECSA-specific activity (Fig. 3e and Supplementary Fig. 12) on the mass activity overwhelmed the that of ECSAs, which were maintained within a deviation of only 4.6% (Fig. 3c). The reduction in the ECSA-specific activities resulted in a noticeably lower mass activity of PWP than that of the WP-structured catalysts (Fig. 3a and Supplementary Fig. 11b). Furthermore, the decrease in mass activities resulting from the change of stacking direction of Ir NW arrays (from perpendicular to parallel) for the denser structures (P200 and P1200 in Fig. 4d, f) became relatively less significant than that for the more open NW building blocks (P400) (Fig. 4e). The opening ratio (OR) of 3D catalysts (ratio of the spacing between NWs divided by NW width), which is in the order of P200 (OR = 0.250) > P1200 (OR = 0.208) > P400 (OR = 0.125), is thought to be inversely scaled with the ease of bubble transport. This is consistent with the considerable loss of ECSA-specific activity by the structural change from WP to PWP in Fig. 3e (indicated with dotted red arrows). In addition, the ECSA-specific activity increases less rapidly with increasing macropore size in the 3D WP samples (Fig. 3e), suggesting that there is an optimal macropore size. For example, the change of building block from P200 to P400 resulted in a 31% increase of ECSA-specific activity, while there was only a 3% improvement by the change from P400 to P1200.

The ECSA-specific activity can be affected not only by the mass transport of the reactants/products of the reaction within the nanostructures but also by the conductivity of the electrons/protons, which can be characterized by the Ohmic loss measured in the EIS analysis at the OER-occurring potential (1.55 V vs RHE) in Supplementary Fig. 10. Regardless of the stacking directions, all NW stacks showed a comparable Ohmic resistance to that of the Ir black samples (Supplementary Table 6). This suggests that in the 3D WP structures, the water inside the well-defined nanoscale pores can provide ionic conductance over a short distance even in the absence of ionomers^38^.

### OER stability

Figure 3f presents normalized current densities over the course of 500 repeating chronoamperometry for the three types of 4-layered 3D WP samples and an Ir black nanoparticle electrode as a reference. The normalized current densities (>0.8) after 500 cycles of the 3D WP samples are comparable to each other, but for the Ir black nanoparticles, the current density decreased by ~40%. The trend of the superior stability of the WP samples was maintained for even more (1000) cycles of the OER (Supplementary Fig. 16a), which was also demonstrated with chronopotentiometry in Supplementary Fig. 17. The decrease in the integrated surface charge during the 500 cycles (inset of Fig. 3f) can be attributed as the dominant factor in the OER stability of the samples. To quantify ECSA, as an alternative for the estimation based on HUPD peaks, we exploited electric double-layer charge calculated from the CV data (Supplementary Fig. 8) and capacitances (Supplementary Fig. 9h) measured with another CV ranging 1.1–1.2 V_RHE_ at various scan rates (Supplementary Fig. 9a–g). In contrast to the significant degradation of the active surface area of the nanoparticle-type catalysts, the WP samples have more capability to sustain an active surface area. It was reported that coalescence or detachment of Ir nanoparticles could lead to a severe loss of surface area (>50%)^39–41^. Agglomeration of Ir nanoparticles also could be expedited by ionomer degradation accelerated by the applied voltage^42,43^.

In contrast, the WP geometry was almost perfectly maintained even after 500 cycles of the degradation test, as shown in Supplementary Fig. 18, although they experienced similar amorphization and hydrated oxidation (Supplementary Fig. 16d–g) of the Ir-based OER catalysts in the acidic electrolyte. This structural robustness suggests that the inherent dissolution of Ir^44,45^ is the main factor underlying the gradual OER performance degradation of the WP structure. However, as shown in Supplementary Fig. 16c, the Ir dissolution rate was relatively smaller for the WP samples compared to Ir black. Previous studies reported that there is an apparent tendency of decelerated Ir dissolution in properly confined nanostructures^11,46^. Also, for our WP samples, the 4-layer 3D geometry having more confined conditions improved the OER stability relative to the 2-layer structure (Supplementary Fig. 16b), suggesting that optimization of the 3D geometry may be able to provide a simultaneous influence on OER activity and stability.

### Characterization of OER performance in PEMWE

**Fig. 5:**
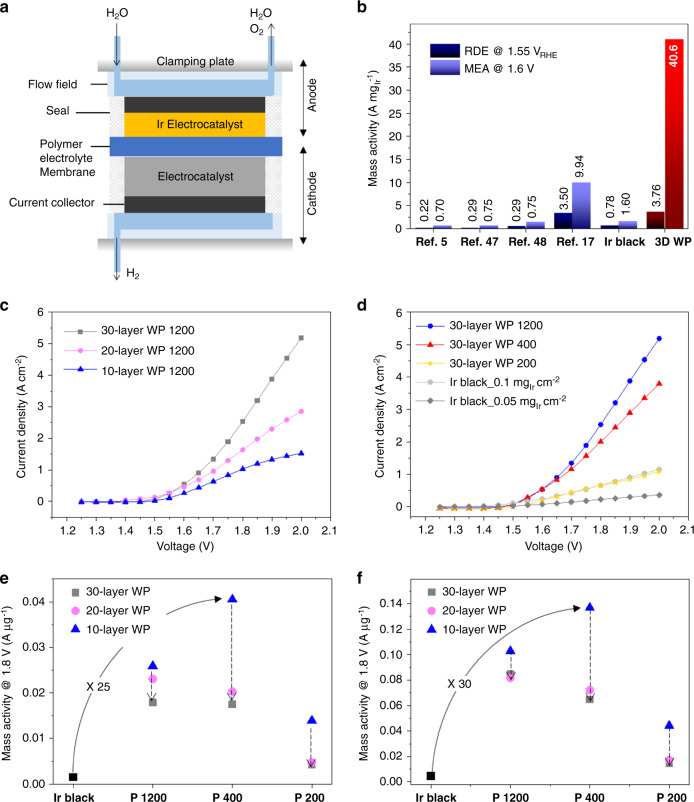
PEMWE performances of 3D WP catalysts. **a** Schematic of PEMWE cells. **b** Comparison of OER mass activities of WP Ir catalysts with those reported in previous studies^5,17,47,48^ based on both half-cell tests using a rotating disk electrode (RDE) and single-cell tests using a membrane electrode assembly (MEA). **c** I–V curves with varying stacking number. **d** I–V curves for three types of building blocks. Comparison of mass activity estimated at **e** 1.6 V and **f** 1.8 V vs. RHE, respectively.

We next demonstrate the outstanding catalytic performance of WP structures when applied to single-cell PEMWE devices. The current density of the single cell (Fig. 5a) containing WP1200 as the anodic catalyst increased as the number of stacking layers was raised from 10 layers to 30 layers, as shown in the I–V curve of Fig. 5c. This can be understood by the linearly enlarging surface area along the number of stacking layers, as confirmed in the half-cell test (Supplementary Figs. 14a and 15). The same tendency was observed for WP400 and WP200 samples (Supplementary Fig. 20). Among the 30-layered WP Ir samples, the current densities were in the order of P1200 > P400 > P200 (Fig. 5d). The 30-layered P1200 sample recorded a maximum current density of 5.2 A cm^−2^ at 2.0 V, which is more than double of the conventionally reported maximum current densities of 1.0–3.0 A cm^−2^ at >1.8 V^7^.

In our setting of single-cell measurements, the WP 1200 30-layered sample showed the maximum current density with an Ir loading of ~45 μg cm^−2^, and the maximum current density was 4.3-times higher than that of the cell based on Ir black nanoparticles (Fig. 5d) with comparable Ir black loading (50 μg cm^−2^). In addition, the PEMWE equipped with 30-layer WP1200 sustained the advanced performance for longer than 45 h as shown in Supplementary Fig. 21.

Moreover, the best membrane electrode assembly (MEA) based on 10-layer P400 catalysts recorded a mass activity (40.6 A/mg) at 1.6 V, which corresponds to 25 times and 17 times the values of the Ir-black-based cells with a comparable Ir loading amount (50 μg cm^−2^) and with the optimum Ir loading (=100 μg cm^−2^) measured in this study (Fig. 5e). The mass activity is approximately four times greater than the highest previously reported value^17^ ($9.94\,{\mathrm{A}}\,{\mathrm{mg}}_{{\mathrm{Ir}}}^{ - 1}$, Fig. 5b) among those of Ir-based catalysts. At 1.8 V, even 30 and 21 times higher enhancement of the mass activity (vs. Ir black nanoparticles having comparable loading and optimized mass activity, respectively) was achieved (Fig. 5f), which suggests that the 3D WP structures allow more efficient transport of O_2_ gas bubbles due to their far more open architecture.

The correlation between the geometric properties of WP structures and the mass activity further validates the critical role of bubble transport in the catalyst. Among the 10-layered WP catalysts, the mass activity was in the order of WP400 > WP1200 > WP200, which is consistent with the half-cell test performed with 4-layered samples. (Fig. 3a) A different trend, however, was observed for the larger number of stacking layers: WP1200 > WP400 > WP200 for 20- and 30-layered WP structures.

The decrease in mass activity accompanied with the addition of more layers was much less significant for P1200 compared to P400 and P200. In the cases of P400 and P200, the mass activity decreased by ~50% from 10 layers to 20 layers and 10–15% from 20 layers to 30 layers at both 1.6 and 1.8 V. In contrast, P1200 showed only a 10–20% decrease of the mass activity upon changing from 10 layers to 20 layers and with additional stacking from 20 to 30 layers. As the path length for transport of O_2_ would be increased for a higher number of stacked layers, while increasing amount of bubbles, the decrease in mass activity accompanied with the addition of layers was much less significant for P1200 having larger channel size compared to P400 and P200. These results indicate that well-defined macropore channels with appropriate size WP structures are critical for high-current density and mass activity, although the optimum structural parameters depend on the operation conditions and the total loading amount of Ir.

## Discussion

In this study, WP-structured Ir catalysts were designed and successfully fabricated via a high-resolution printing process to enable extremely high mass activity in the OER, which is driven by both high ECSA and ECSA-specific activity. A half-cell test demonstrates that the WP catalyst generated 4.8-times higher mass activity than the Ir black nanoparticles despite no difference in the physiochemical properties. More strikingly, when applied to a single-cell PEMWE device, the new catalyst achieved far more enhanced mass activity, up to 30 times that of Ir black. Systematic tuning of the 3D geometry and NW building blocks revealed that O_2_ bubble transport, which was characterized with SECM, is a key parameter in improving the performance of the OER catalysts, providing practical design rules for more efficient OER catalysts. We expect that these outcomes will provide a large step toward the development of highly feasible OER catalysts by simply controlling their 3D microstructures even without any change of physiochemical characteristics. Therefore, there will be abundant room for further improving the OER performance of our WP-structured catalysts via synergistic combination with other approaches based on new elemental compositions.

## Methods

### Fabrication of WP-structured Ir thin film

Prior to the S-nTP process, we prepared three types of line-patterned master templates with periods of 200 nm, 400 nm, and 1 μm via photolithography and plasma etching. A polydimethylsiloxane (PDMS) brush (PDMS-OH, molecular weight = 5 kg/mol) with a low surface energy was grafted on the surface of the master templates. On top of the brush-treated mater template, a polymethylmethacrylate (PMMA) was spin-coated to produce a polymer replica, which was detached using a polyimide film. On the PMMA replica, an oblique angle deposition process was employed through e-beam deposition to create discrete Ir NWs on the replica. The NWs were transfer-printed onto a Cu receiver substrate, and then the polymer replica was removed by toluene washing. Sequential printing of Ir NWs was then repeated, forming multilayer stacks of 3-dimensional structures, as illustrated in Fig. 2. The NWs arrays were then transferred to a working electrode for the half cell test or the Nafion^®^ membrane for the single-cell test. To transfer Ir NWs from the receiver substrate onto a glassy carbon electrode or Nafion membrane, the Cu substrate was removed with a commercialized Cu etchant (Sigma Aldrich, Ammonium Persulfate). After etching the Cu foil, the nanostructured Ir samples floating on the surface of the Cu etchant were lifted up with the substrates for electrochemical measurements. To measure the loaded amount of Ir in each sample, inductively coupled plasma-mass spectrometry (ICP-MS) measurements were performed at least five times.

### Physiochemical characterization

The physiochemical characteristics of the Ir OER catalysts were analyzed with various methods including XRD, TEM, XPS, and X-ray absorption fine structure (XAFS). XRD measurements were performed on a SmartLab (RIGAKU) diffractomer in θ–2θ scan mode using Cu *K*_*a*1_ radiation generated by high power (>9 kW) and a symmetric-cut curved crystal monochromator. A TEM analysis was carried out using JEM-2100F (HR) (JEOL LTD., Japan)) with an acceleration voltage of 200 kV. XPS measurements were performed using a K-alpha spectrometer (Thermo VG Scientific) with a microfocused monochromatic X-ray source. The reference of the binding energies of the acquired spectra was the C 1 s line at 284.8 eV. XANES and EXAFS were collected at the 1D XRD KIST_PAL beam line. The incident beam was collimated with a mirror of Rh/Pt, two Ag-coated strips, and monochromatized using a double crystal monochromator composed of in situ Si(111) and Si(311) exchange. The monochromatized beam was refocused with a focusing mirror (Ag-coated strips, Si-cylindrical, and Rh/Pt coated). The photon flux of the beam was measured with an ionization chamber equipped with a 1000 V-biased Ni electrode. EXAFS data were calibrated with energy background subtraction and edge step normalization. The resulting spectra were converted to the photoelectron wave vector k, and the resulting χ(k) functions were weighted with *k*^2^ to compensate for the dampening XAFS amplitude with increasing k.

### Electrochemical analysis in half cell

The electrochemical characterization of WP-structured Ir and Ir black nanoparticles (Premetek Co., product number P40V010) was performed in a half-cell setup equipped with a glassy carbon working electrode, platinum counter electrode, and a reversible hydrogen electrode (RHE). Working electrode rotation was controlled with a modulated speed rotator (Pine Instruments, U.S.) and electrochemical measurements were taken with Interface 1000E potentiostat (Garmry, USA). The commercial Ir black catalysts were prepared as a suspension ink using 3.5 mg of catalyst, 2.0 mL of deionized (DI) water, 8.0 mL of isopropyl alcohol, and 40 μL of Nafion^®^ (5 wt%, Sigma Aldrich) solution. The ink suspension was sonicated for 30 min before pipetting 10 μL onto a polished glassy carbon disk electrode. The area of the glassy carbon electrode was 0.196 cm^2^ and thus the Ir electrode loading of Ir black corresponds to 17.8 μg/cm^2^. The composition of the ink was optimized based on the mass activity for Ir loading and Nafion^®^ content. For the stack of WP NW arrays, the amount of Ir loading and the thickness were adjusted by controlling the number of stacked layers.

All potentials reported herein refer to the RHE scale and have been corrected for the Ohmic drop in solution. The 0.05 M H_2_SO_4_ electrolyte was prepared from 98% H_2_SO_4_ (Sigma Aldrich) and was saturated with synthetic air during all measurements. Following 5 cycles of cyclic voltammetry (CV) ranging 0.05–0.6 V for electrochemical reduction of surface iridium oxide, CVs ranging 0.05–1.4 V was conducted at 50 mV/s until the metallic Ir on the surface of the catalyst had been transformed to a hydrated Ir oxide film (HIROF), which is indicated by the disappearance of the HUPD peak. Linear polarization curves were completed in a potential range of 1.2–1.7 V at 5 mV/s and a rotation speed of 2500 rpm. Catalyst activities were evaluated at 1.55 V. Mass activities were defined as the OER activity normalized to the mass of Ir loaded on the working electrode. The ECSA-specific activities were defined as OER activities normalized by the ECSA. The ECSA of each sample was derived by integration of the current response (normalized to the substrate geometric surface area) from the CV in a potential range of 1.0–1.4 V. In this voltage range, there is no peak caused by the kinetic polarization and the current density of the flat region increases linearly with a scan rate 10 mV s^−1^. Stability of WP catalysts was evaluated with repeating chronoamperometry, which was performed by stepping between an open circuit potential (1.00 V), where no OER current is observed, and a potential (1.6 V_RHE_: an overpotential of 0.27 V from the equilibrium potential of 1.23 V_RHE_), where an appreciable OER current is obtained. Each potential was maintained for 10 s. During 500 cycle of the stability test, the 0.5 mL of the electrolyte was collected every 50 cycles, and ICP-MS analysis was applied to measure the concentration of Ir dissolved from the electrode to the electrolyte. All reported measurements were repeated at least three times to ensure reproducibility. The error bars in the plots indicate the standard deviation of the measurements.

### Scanning electrochemical microscopy

The SECM tip was a Pt-disk microelectrode with a diameter of 25 μm. An Ag/AgCl electrode was employed as a reference and the counter electrode was a Pt-wire. Before each measurement, the microelectrode was polished with 0.3 μm alumina paste, cleaned by ultrasonication in a deionized water bath, and then rinsed with deionized water.

SECM experiments were performed by using a four-electrode system consisting of CE, RE, and two WEs (tip and sample) controlled by a bipotentiostat (CHI 920D). The position of the tip was adjusted in the *x*, *y*, and *z* directions by a stepper motor-based SECM system.

The SECM tip was placed 7 μm away from the surface of the catalyst electrode, confirmed by SECM feedback mode. The potential applied to the tip was −0.05 V (reduced enough to collect evolved O_2_), while the WP and WP samples were polarized in the potential range between 1.1 and 1.4 V_Ag/AgCl_.

### Single-cell measurements

MEAs were fabricated and tested to demonstrate the performance of a single cell when the WP Ir samples were applied as the anode catalyst. An MEA was prepared by loading WP Ir on the Nafion^®^ 212 as the anode catalyst. The WP samples were prepared with three types of building blocks, P1200, P400, and P200, varying the stacking number (10, 20, and 30). For comparison, for an MEA using Ir black nanoparticles (Premetek Co., product number P40V010) as the anode catalyst, the nanoparticle slurry was spray-coated on the membrane. The cathode was prepared by spraying the catalyst mixture, consisting of isopropyl alcohol, water, Nafion^®^ ionomer, and Pt/C 46.2 wt%, on 10 BC (SIGRACET®, product number 10 BC, 385 ± 50 microns in thickness). Pt/C catalysts with a loading amount of 0.4 mg_pt_ cm^−2^ were loaded with 30 wt% Nafion^®^ content. A nanoparticle-type Ir black anodic catalyst was spray-coated on the Ti GDL (Bekaert, 250 μm) with a loading amount of 0.1 and 0.05 mg cm^−2^ with 10 wt% Nafion^®^ content at the opposite site of the cathode. Hot pressing was performed at 140 °C for 2 min to combine the prepared cathode and anode as a device. The MEAs were characterized with a high-current potentiostat (HCP-803, Bio-Logic). The cell was operated at 80 °C with water fed to the anode at a rate of ~15 mL min^−1^ without back pressure. I–V curves were plotted with the average current measured for 1 min at 0.05 voltage increments in the range of 1.25–2.0 V. Durability of PEMWE loading WP1200 structures as anode was tested at current density of 100 mA cm^−2^.

## Supplementary information

Supplementary Information

## Data Availability

Data supporting the plots and other findings in this article are available from the authors upon reasonable request.
