# Supplementary material for: Highly efficient oxygen evolution reaction via facile bubble transport realized by three-dimensionally stack-printed catalysts
Source: Nat Commun. 2020 Oct 1;11:4921. doi: 10.1038/s41467-020-18686-0 (PMC7529785; doi:10.1038/s41467-020-18686-0)
Supplement: Supplementary file 1 — Supplementary Information [file 41467_2020_18686_MOESM1_ESM.pdf]

Supplementary Information

Highly efficient oxygen evolution reaction via effective bubble transport realized by three-dimensionally stack-printed catalysts

Kim et al.

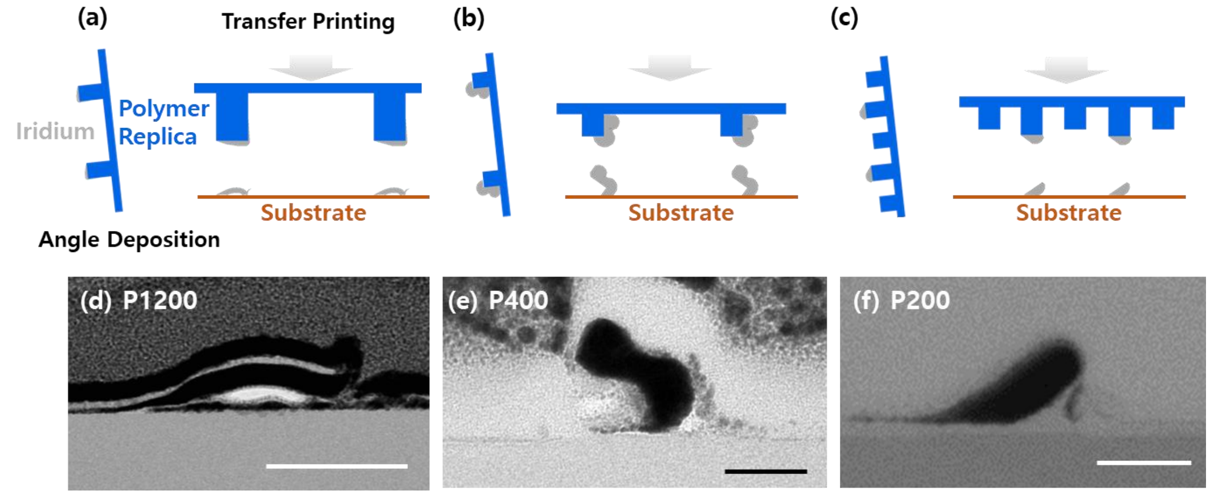

Supplementary Figure 1| TEM images of cross-sectioned nanowires used as a building block in the woodpile structures. The schemes describe nanowire (NW) cross-sections formed through solvent-assisted nanotransfer printing (S-nTP) in woodpile (WP) structures: (a) WP 1200, (b) WP 400, and (c) WP 200. TEM images present the nanowire cross-sections after printing and FIB-sectioning of (d) WP 1200, (e) WP 400, and (f) WP 200 samples, respectively. Scale bar in (d) indicates 100 nm, and scale bars in (e) and (f) are 30 nm.

Supplementary Table 1|Comparison of Ir-based loading amount for building blocks fabricated by S-nTP

(The loading amount of Ir per 1 layer of building block on 1 cm^2($Amount_{Ir,cal}$), the calculated specific surface area (CSA), and the utilization efficiency were calculated based on the *Image J* analysis on the area and perimeter of the cross-sectioned nanowires, following equations:

$\mathrm{Amount_{Ir,cal}}$ = total volume of Ir NWs per area × density of Ir $= \frac{1\ \mathrm{cm}}{\mathrm{p_{NW}}} \times 1\mathrm{cm} \times \mathrm{a_{NW}} \times$ density of Ir, $\mathrm{CSA} = \mathrm{s_{NW}} \times 1\ \mathrm{cm} \div \frac{1\ \mathrm{cm}}{\mathrm{p_{NW}}} \div \mathrm{Amount_{Ir,cal}}$, Utilization Efficiency $= \mathrm{ECSA_{cal}} \div \mathrm{ECSA_{meas}}$. Here, $\mathrm{a_{NW}}$ [$\mathrm{m^2}$], $\mathrm{s_{NW}}$ [m], and $\mathrm{p_{NW}}$ [cm] are area of a $\mathrm{NW_{cross-sectioned}}$, perimeter of a $\mathrm{NW_{cross-sectioned}}$, and period of NW array, repectively)

Building Block	Amount of Ir [$\mu g\ cm^{-2}$]		Surface Area [$m^2\ g^{-1}$]		Utilization Efficiency
	Calculation	Measurement	CSA	ECSA	
P1200	1.07	1.00	41.7	40.9	98.1%
P400	1.16	1.03	44.1	43.6	98.9%
P200	1.66	1.49	35.8	33.8	94.4%

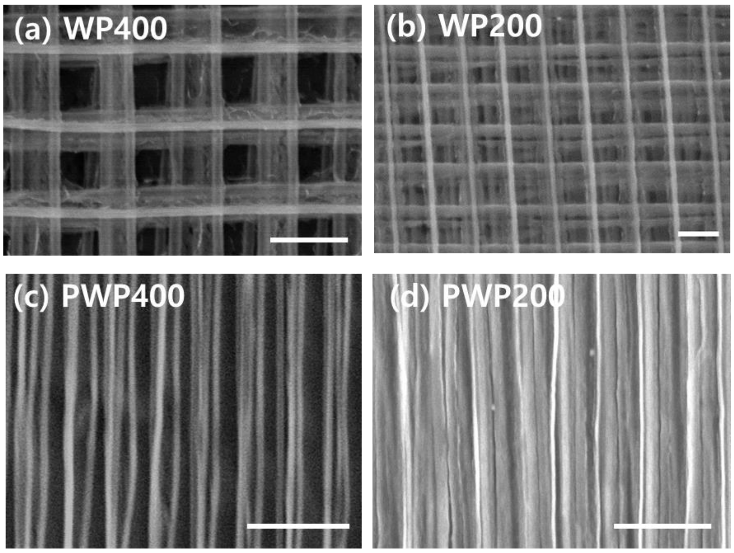

Supplementary Figure 2| Scanning electron microscopy (SEM) images. (a) 10-layer WP400, (b) 10-layer WP200, (c) PWP400, and (d) PWP400. Scale bars are 400 nm, 200 nm, 1μm, and 500 nm in the order from (a) to (d).

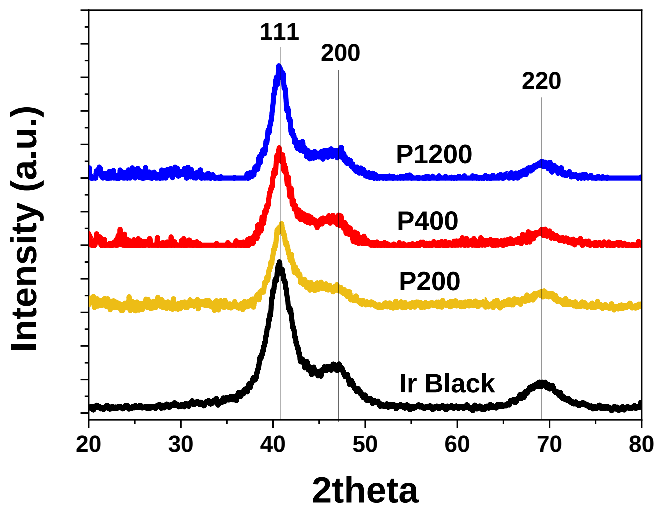

Supplementary Figure 3| X-ray diffraction (XRD) patterns. There was no difference in the crystal structure of iridium for all three nanowires despite their various shapes, having common dominant facets within face-centered cubic structure of iridium metal.

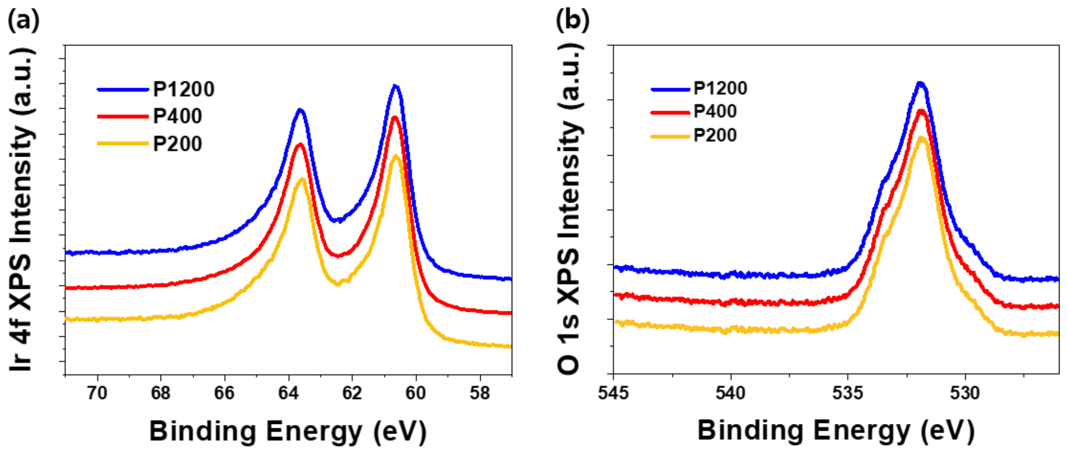

Supplementary Figure 4| Ir 4f XPS peak analysis. (a) Ir 4f spectra and (b) O 1s spectra of nanowires fabricated by S-nTP, where peaks exist at negligibly different positions with analogous ratios of their intensity.

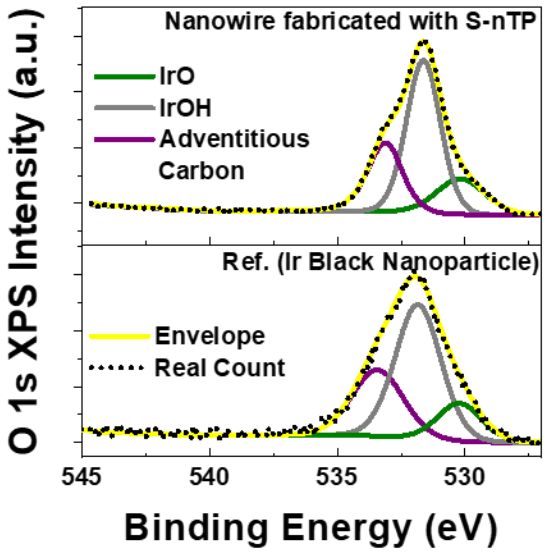

Supplementary Figure 5| O 1s XPS peak analysis. O 1s spectra indicating the peak position from iridium oxide (green), iridium hydroxide (grey), and adventitious carbon species (purple), which reveals the surface oxidation state of the samples. The oxide and hydroxide species of Ir can be observed at approximately 530 and 531 eV, respectively.[1,2] The low, broad features existing near 533 eV are assigned to the presence of a small fraction of oxygen components from adventitious carbon species.[2] Although the O 1s spectra are also affected by other components, we can posit similar oxidation states of iridium between the printed NWs and commercial Ir black nanoparticles.

Supplementary Table 2| Quantification results of XPS O 1s peak

	Ir Black		S-nTP Nanowire	
	Position [eV]	%	Position [eV]	%
IrO	530.51	32.8	530.17	32.6
IrOH	532.06	34.0	531.63	34.4
Adventisious Carbon	533.7	33.2	533.31	33.0

Supplementary Table 3| Quantification results of XPS Ir 4f peak

(In IrO_2, photoemission of Ir $4f_{7/2}$ ($4f_{5/2}$) electrons gives rise to the intensity at 61.8 eV (64.8 eV) with an anomalous, highly asymmetric line shape[3], and amorphous IrO_x has the intensity at around 62.4 and 65.4 eV. Ir(III) comes from amorphous IrOx.)

	Ir Black		S-nTP Nanowire	
	Position [eV]	%	Position [eV]	%
Ir metal	60.99	32.1	60.93	32.2
Ir(IV)	61.68	32.0	61.31	32.4
Ir(III)	62.18	35.9	61.9	35.3

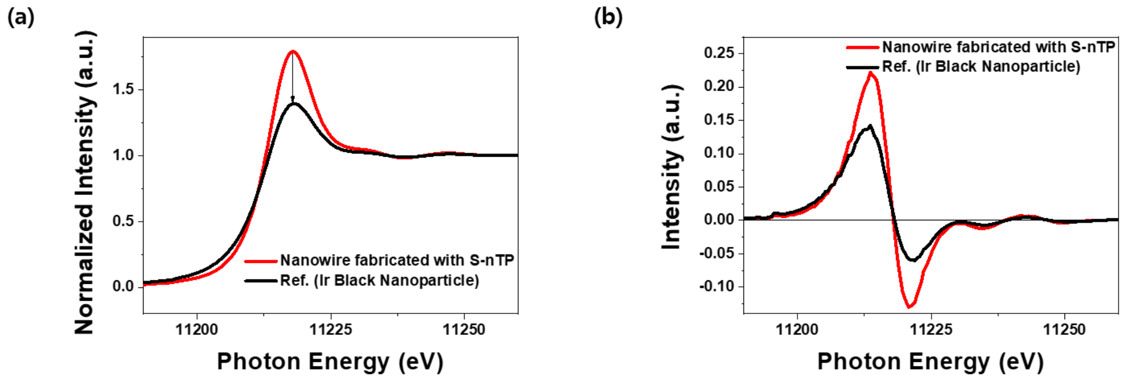

Supplementary Figure 6| XAS Analysis. (a) XANES spectra and (b) First derivative of XANES spectra. The difference in the L_{III} edge peak magnitude between the printed nanowires and the Ir black nanoparticles is typically representative of differences within the d-band structure of the iridium atoms. The white line (WL) peak at the L_III-edge is caused by transitions from 2p to empty localized 5d states, which is localized to the atoms on the surface.[4,5] The edge energies were determined from the first derivatives of μ(E) (Fig. S3b). The values were between 11217.9 eV (for P200) and 11218.17 (for Ir Black), and these are within the error range (±0.4 eV) of the 1D XRD KIST_PAL beam line. The samples with a larger ratio of surface-to-bulk atoms tend to be more sensitive to the transition because XAS is bulk sensitive,[6] implying a larger surface area of the nanowire stacks fabricated through S-nTP than the agglomerated nanoparticles. This phenomenon corresponds to higher intensity in the XANES data of the nanowires fabricated with S-nTP than that of Ir Black nanoparticles; the stack of nanowires fabricated by S-nTP showed a larger ECSA than the Ir Black nanoparticles.

Supplementary Table 4| Parameters estimated from EXAFS data

(Coordination number (N) of the given scattering path, radial distance (R) of bonding in nanowire fabricated through S-nTP and nanoparticle Ir Black, Debye-Waller Factor (σ^2), and energy shift (E_o) were calculated by fitting of EXAFS intensity with the two-cluster model. The two-cluster model includes the six nearest neighboring oxygen atoms at ~1.999 Å and the next nearest neighboring iridium atoms at ~3.159 and ~3.556 Å with the distance, Debye-Waller factor, and coordination number being refined in the fit. The model captures all of the features of the iridium local environment up to 4Å.)

Samples	E_0(eV)	Shell	N	R (Å)	σ^2 (Å^2)
**Nanowire Fabricated with S-nTP**	13.05	Ir-O	2.2±0.4	1.97±0.01	0.003
		Ir-Ir	11.6±1.8	2.697±0.004	0.003±0.001
		Ir-Ir	4.1±2.9	3.84±0.05	0.005
**Ref. (Ir-Black Nanoparticle)**	12.55	Ir-O	2.9±0.6	1.98±0.01	0.005±0.003
		Ir-Ir	8.0±0.8	2.696± 0.005	0.004±0.001
		Ir-Ir	3.3±2.6	3.83±0.04	0.005±0.005

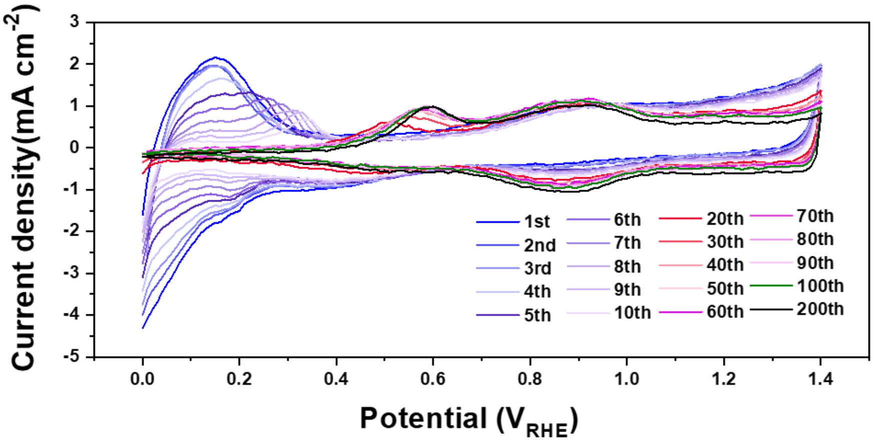

Supplementary Figure 7| Cyclic Voltammetry for irreversible electrochemical oxidation. The cyclic voltammetry (CV) of 10-layer WP P1200 was conducted with a scan rate of 100 $mV\ s^{-1}$ under N_2 purging, and the same protocol for electrochemical oxidation were adopted to all samples tested in this study. The peak of hydrogen underpotential deposition in the first cycle of the CV was exploited for measurement of the electrochemically active surface area (ECSA).

Supplementary Table 5| ECSAs and their utilization efficiency of the Ir black nanoparticles

(The calculation of ECSA, which means theoretical specific surface area, was conducted with the following equation: $\mathrm{ECSA} = \frac{4\pi(\text{radius of Ir NP})^2}{(\text{Density of Ir})\times\frac{4\pi}{3}(\text{radius of Ir NP})^3}$)

Diameter of Ir Nanoparticle	Surface Area $[m^2\ g^{-1}]$			Utilization Efficiency
	Calculation (CSA)	Information from manufacturer	Measurement (ECSA)	
3.4 nm (average calculated from XRD peak)	115.3	55-65	55.0	47.7%

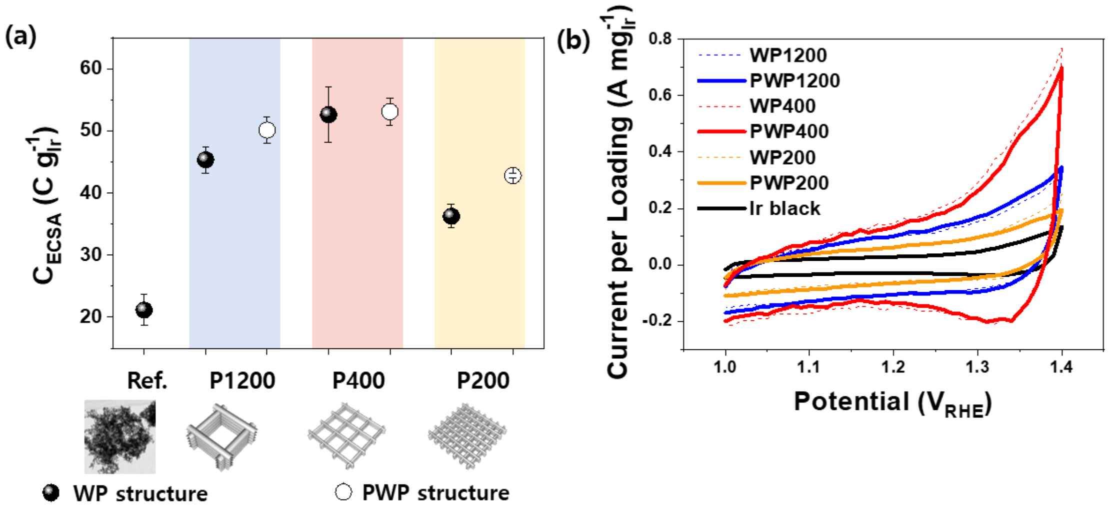

Supplementary Figure 8| Indirect estimation of ECSA with electric double-layer charge. (a) Electric double-layer charge was calculated from integration of the positive current densities in (b) CV with a scan rate of 10 mV s^{-1} at the non-Faradaic region after normalization with the Ir loading amount. 1.0 – 1.4 V_{RHE} range of the CV was a 0.1 V potential window centered at the open-circuit potential (OCP) of the system: 1.1 - 1.35 V_{RHE}. We observe the consistency of the electric double-layer charge with electric double-layer charge capacitance measured from the non-Faradaic capacitive current in the CVs (between 1.0 and 1.2 V_{RHE}).

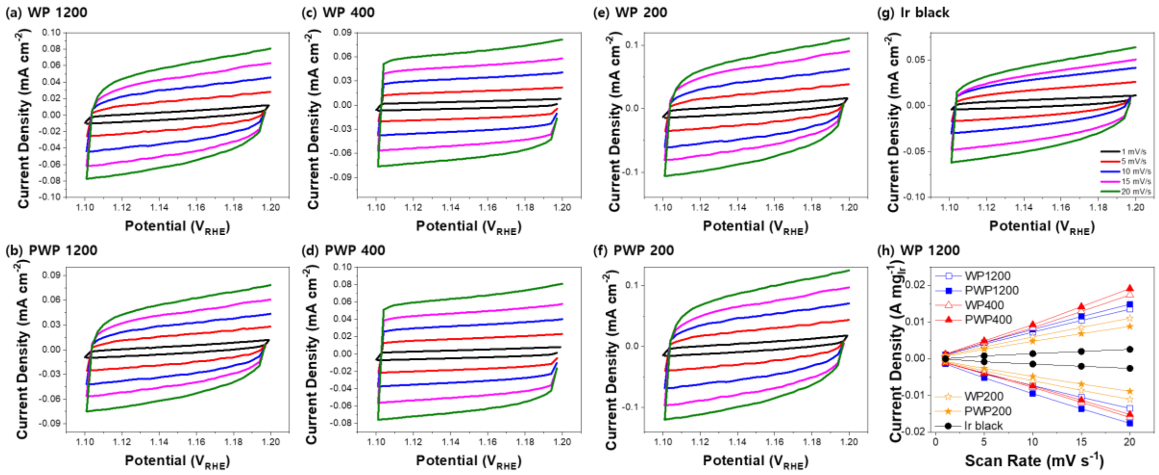

Supplementary Figure 9| Electric double-layer Capacitance. (a-g) Cyclic voltammetries ranging 1.1 – 1.2 V_{RHE} at various scan rates. The catalyst capacitance normalized by the loaded iridium was estimated from the slope of (h) the current densities plotted as a function of scan rate: 0.652 F mg_{Ir}^{-1}, -0.652 F mg_{Ir}^{-1} (WP 1200), 0.718 F mg_{Ir}^{-1}, -0.848 F mg_{Ir}^{-1} (PWP 1200), 0.854 F mg_{Ir}^{-1}, -0.783 F mg_{Ir}^{-1} (WP 400), 0.940 F mg_{Ir}^{-1}, -0.744 F mg_{Ir}^{-1} (PWP 400), 0.535 F mg_{Ir}^{-1}, -0.535 F mg_{Ir}^{-1} (WP200), 0.428 F mg_{Ir}^{-1}, -0.428 F mg_{Ir}^{-1} (PWP 200), 0.131 F mg_{Ir}^{-1}, -0.131 F mg_{Ir}^{-1} (Ir black).

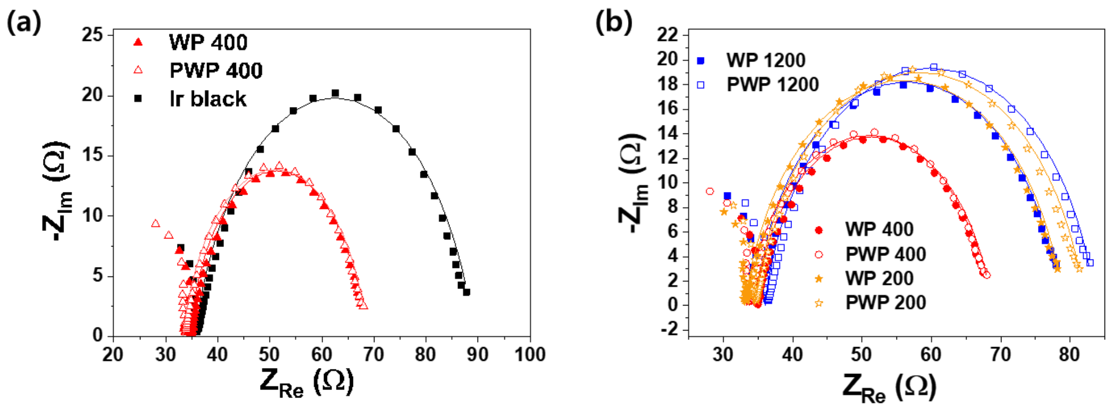

Supplementary Figure 10| Electrochemical Impedance Spectroscopy. Nyquist plots for (a) Ir Black nanoparticles and the P400 NW arrays stacked in the parallel/perpendicular direction and (b) the three different types of Ir NW arrays stacked in parallel/perpendicular direction. They were measured in 0.5 M H_2SO_4 at 1.55 V vs RHE measured from Electrochemical Impedance Spectroscopy (EIS) in a frequency range of 100 kHZ to 100 mHz. In this potential, the oxygen evolution reaction can be observed. The solid lines are fitted to the data using the simplified Randles circuit.

Supplementary Table 6| Ohmic resistance calculated from EIS data at 1.55 V vs RHE

Samples	R_{Ohm} [Ω]	Error
Ir Black	35.8	0.15
WP 1200	34.5	0.092
PWP 1200	36.1	0.12
WP 400	34.8	0.13
PWP 400	33.5	0.13
WP 200	33.3	0.11
PWP 200	34.3	0.26

Supplementary Table 7| Mass activities measured with various loading amounts of Ir for woodpile structured samples and Ir black nanoparticles

Test Type	Sample Name	Loading of Ir [μg cm^{-2}]	Current Density [mA cm^{-2}]	Mass Activity [A mg^{-1}]
Half Cell	Ir Black Nanoparticle	17.8	14.0 (@ 1.55 V_{RHE})	0.79
	WP1200_4 layer	4.00	14.3 (@ 1.55 V_{RHE})	3.58
	WP400_4 layer	4.13	15.5 (@ 1.55 V_{RHE})	3.76
	WP200_4 layer	5.96	11.6 (@ 1.55 V_{RHE})	1.95
Single Cell	Ir Black Nanoparticle	50, 100	80.3, 245 (@ 1.6 V)<br>225, 661 (@ 1.8V)	1.61, 4.90<br>2.25, 6.61
	WP1200_10 layer	10.1	259 (@ 1.6 V)<br>1.03×10^3 (@ 1.8V)	25.9<br>103
	WP400_10 layer	10.33	419 (@ 1.6 V)<br>1.42×10^3 (@ 1.8V)	40.6<br>137
	WP200_10 layer	14.9	208 (@ 1.6 V)<br>659 (@ 1.8V)	14.0<br>44.2

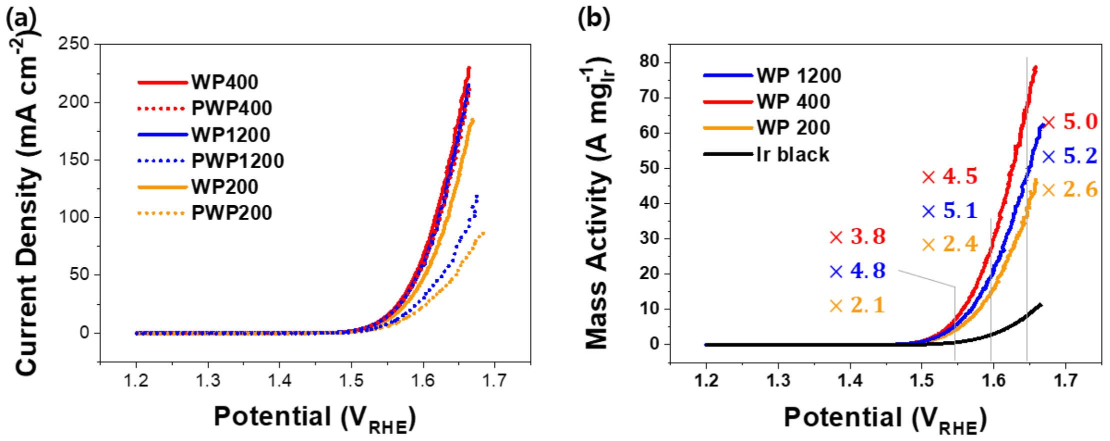

Supplementary Figure 11| Linear sweep voltammetry of current density and mass activity. (a) Linear sweep voltammetry of WP and PWP samples and (b) mass activities of 4-layered woodpile structured Ir thin films and Ir Black nanoparticles.

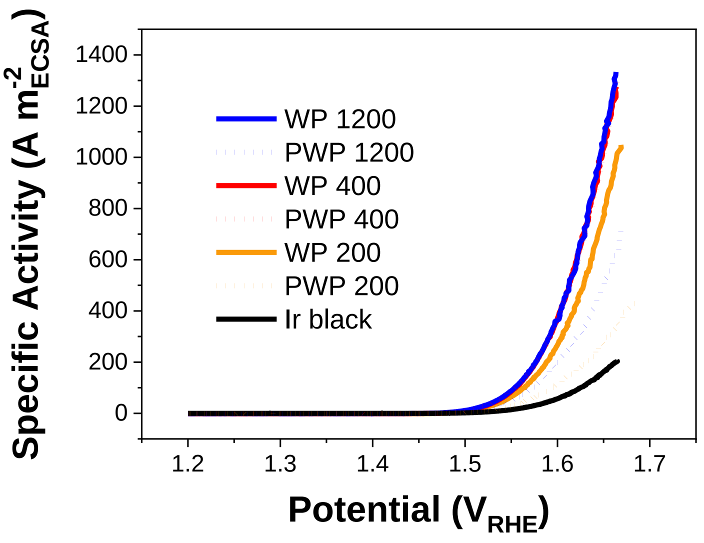

Supplementary Figure 12| ECSA-specific activity. Linear sweep voltammetry of current densities normalized by the loaded amount of Ir in the electrodes and ECSAs. The order of specific activity among samples sustained the trend pointed out at 1.55 V_{RHE} in Fig. 3e

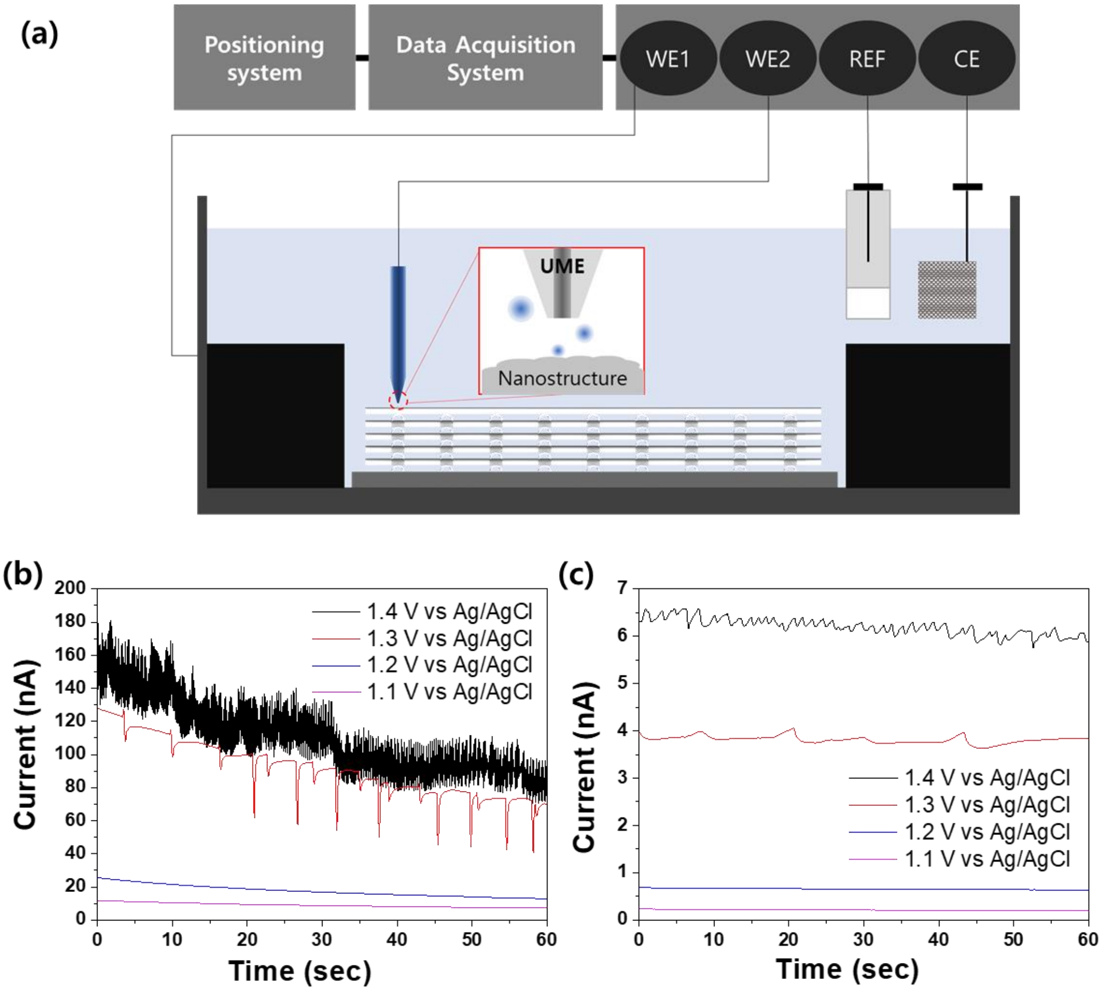

Supplementary Figure 13| Analysis with scanning electrochemical microscopy. (a) Scheme of the scanning electrochemical microscopy (SECM) setup. CE, WE, REF, and UME refer to the counter electrode, working electrode, reference electrode, and ultramicroelectrode, respectively. Current measured using an SECM tip at different applied sample potentials (from 1.1 to 1.4 V_(Ag/AgCl)) of (b) WP and (c) PWP 1200 samples. It is noteworthy that the difference in the frequency of SECM peaks of the WP and PWP structures is more prominent at the higher potential range, where efficient transport of O_2 bubbles becomes more critical due to faster generation of O_2. Analogously, the difference in the mass activity between the 3D WP catalyst and the Ir black nanoparticles rapidly rose with the increase of potential, where the O_2 bubble transport issue would be aggravated.

Supplementary Note 1| Mobility of Nanobubbles in Nanostructured Catalysts

We assume that a nanobubble is generated in nanoporous media. To escape the nanostructure, the nanobubble motion is driven by a buoyancy force within the nanoporous media. Simultaneously, the porous structure provides a restriction to retard the bubble movement and it is posited that the surface tension effect at the wetting area between the bubbles and the catalytic surface holds the bubble.

Here, if the radius of the bubble (r) is extremely smaller than the pore size, the bubble can be considered as a particle when the bubble is detached from the surface of the catalytic structure. In this case, using Darcy's law, the movement of the bubble can be considered as a simple mass transport in a porous medium. Its mean velocity in the porous medium (υ) can then be described as $\upsilon \simeq \frac{r_h^2}{\alpha\mu}\frac{\Delta P}{L}$. $\Delta P/L$ is the pressure gradient (driving force of the buoyancy effect), μ is the dynamic viscosity of the electrolyte, r_h is the hydraulic radius, α is a geometric factor, and L is the channel length. According to the simplified scheme of the pore geometry within the WP and PWP structures, the hydraulic radius can be estimated as follows: $r_h = \frac{Area}{Perimeter}$.

The geometric factor (α) relies on the cross-sectioned shape of pores: $\alpha = 2$ for the WP structure having a square cross-section and $\alpha = 3$ for the PWP structure having a triangular cross-section. The relative mass transport rate can then be estimated as $\frac{\upsilon_{WP}}{\upsilon_{PWP}} = \frac{r_{h,WP}^2}{r_{h,PWP}^2} \times \frac{\alpha_{WP}}{\alpha_{PWP}}$, where we assume the driving force and the viscosity are the same. Here, we use l (= 15 μm) and w (= $\frac{1}{2}p_{g,WP1200} = 950$ nm), measured in the SEM image of Fig. 1h, and the relative mass transport rate approximates to 5.

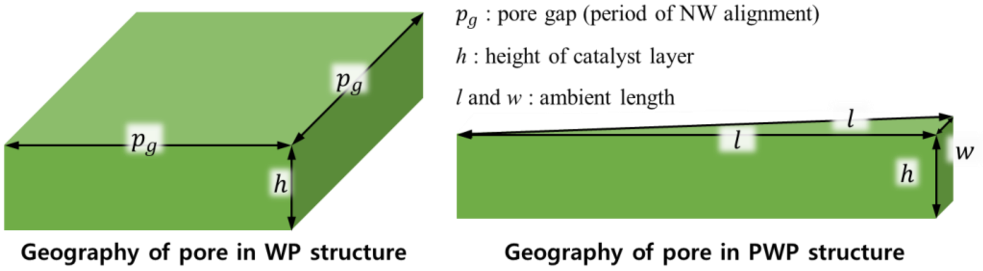

Simplified Scheme of pore within WP and PWP structures

Supplementary Note 2| Relative Frequency of Bubble Formation and Removal from Catalyst Layer

If we assume that the bubble size is comparable with the pore size of the catalyst structure, the wetting condition at the surface of the catalyst that retards the mobility of the bubble should be considered. The surface adhesion force varies with the geometry (stacking direction) of the nanowire arrays, according to numerical investigations of the adherence energy of the capillary bridge at the junction of wires.[7,8] The literature shows that the capillary bridge between parallel fibers has a higher adherence force than the crossing fibers. The capillary bridge and fibers correspond, respectively, to bubbles having size similar to or larger than pores and nanowires forming WP or PWP structures.

It was possible to compare the surface adherence force based on the surface area of the typical pores of WP and PWP structures (Figure S14) because the pore geometry approximates to the interface where the bubbles have physical contact with the catalytic surface. The surface adhesion force (F) is calculated as the product of intrinsic surface tension (γ) and the perimeter (ℓ) of the geometries; i.e., $F = \gamma\ell$, where $\ell_{WP} = (8p_g + 4h)$ and $\ell_{PWP} = (p_g + 4l + 3h)$ based on Figure S14. Here, to estimate the characteristic length scales in the PWP structure, we obtain the typical length l by using the same void fraction (porosity) in two porous structures. Therefore, if the void fraction in the WP and PWP structure is the same, we can obtain $p_g^2 h = \frac{1}{2} lwh = \frac{1}{4} l p_g h$ (w was measured as $\frac{1}{2} p_g$ in the SEM image in Figure 1h). Finally, we can obtain $l = 4p_g$.

In order to remove the bubble from the catalyst layer, the surface force should be smaller than the buoyancy force applied to a bubble having volume V, which can be calculated as $\Delta\rho V g$ (g is gravity acceleration). Considering the force balance between the buoyancy and surface adhesion force, the critical volume (V_c) to be removed from the catalyst layer can be estimated as

$$\Delta\rho V_{WP} g \gtrsim \gamma\ell_{WP} \text{ and } \Delta\rho V_{PWP} g \gtrsim \gamma\ell_{PWP}.$$

If we assume $\frac{h}{2p_g} \ll 1$ and $\frac{3h}{17p_g} \ll 1$, we can obtain the critical volume for each case, such as

$$\therefore V_{WP} \gtrsim \frac{8p_g\gamma}{\Delta\rho g} \text{ and } V_{PWP} \gtrsim \frac{17p_g\gamma}{\Delta\rho g}.$$

From this result, the critical volume V_{PWP} to detach from the catalyst surface is larger than V_{WP}. As a consequence, more bubbles in the WP nanostructure escape frequently.

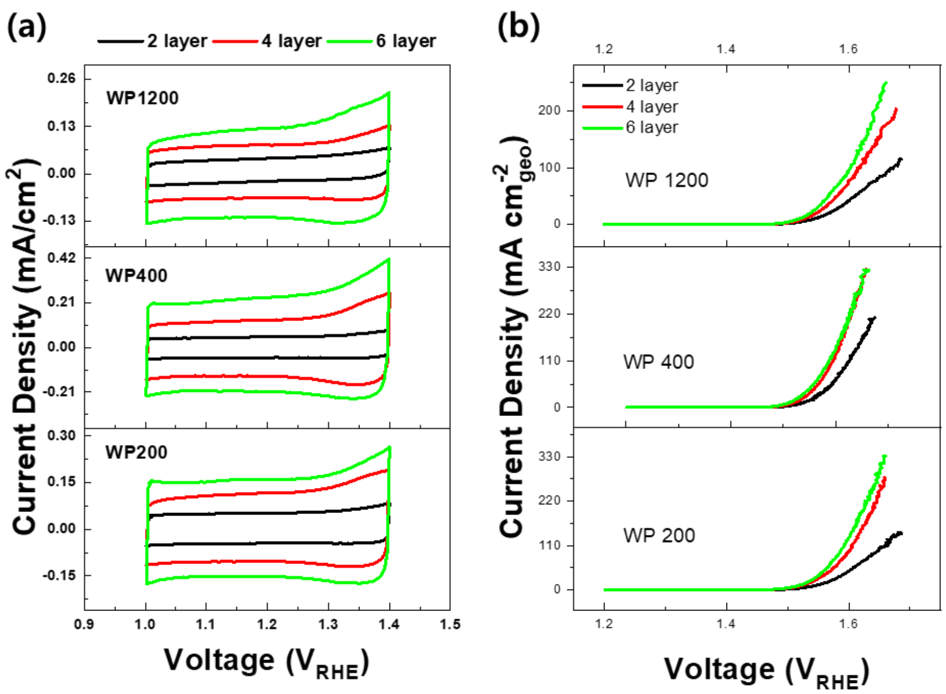

Supplementary Figure 14| Half-cell analysis on the increasing number of layer in WP structures. (a) Cyclic Voltammetry; No decrease in surface area with increasing loaded amount and propensity to flood from top to bottom of the catalyst layer. (b) Linear sweep voltammetry with increasing loading amount.

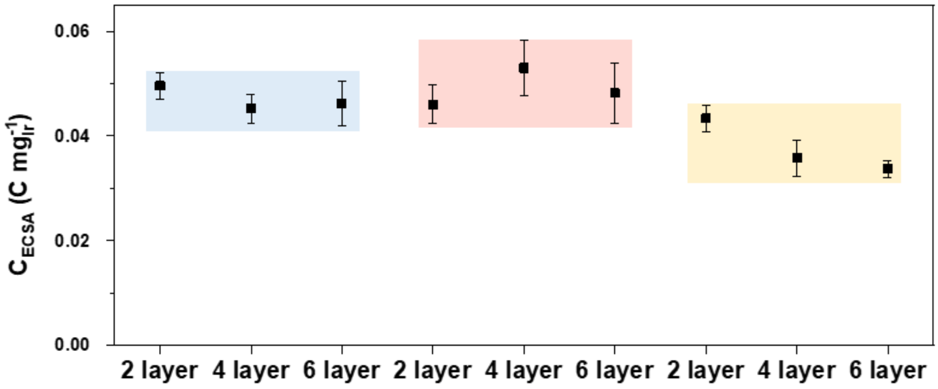

Supplementary Figure 15| Electric double-layer charge calculated from CV ranging 1.0 - 1.4 V_{RHE}. The integrated surface charge (C_{ECSA}), implying the ECSA, was maintained within a certain range in the case of P1200 and P400 despite the change in the number of stacking layers. In the case of P200, although the integrated surface charges tend to decrease with an increasing number of stacking layers, they are within the error range (standard deviation of the measurements indicated as error bars). Maintenance of the ECSA in spite of the increasing loading amount means the surface area of the interface between the catalyst and the electrolyte increases proportionately with increasing loading amount of the catalyst.

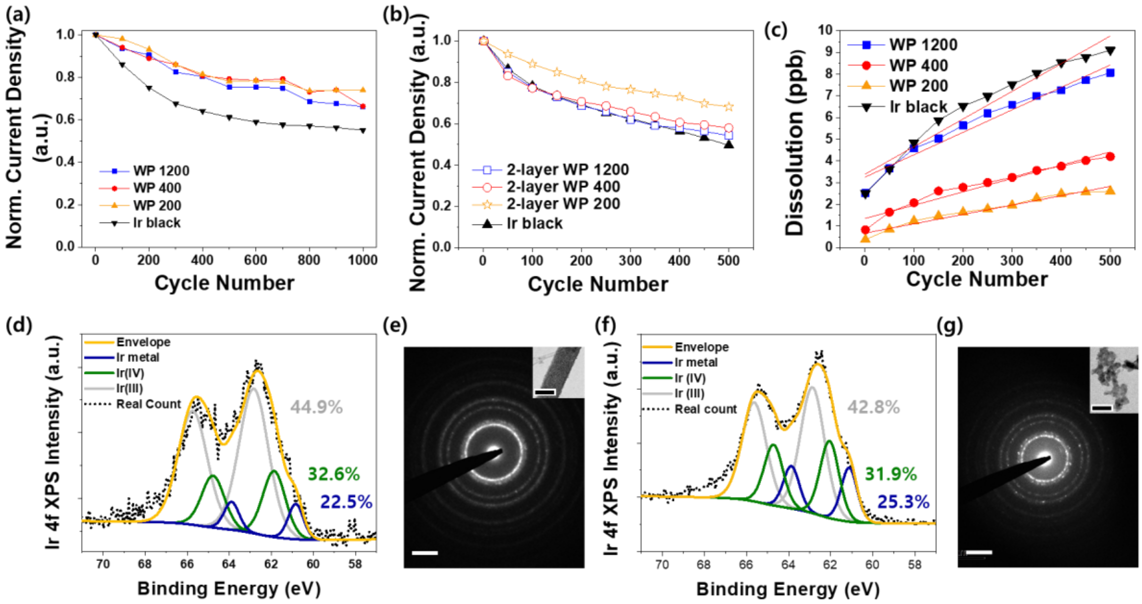

Supplementary Figure 16| Analysis for Stability. Normalized current density during repeating chronoamperometry of (a) 1000 cycles for 4-layer WP samples and Ir Black and (b) 500 cycles for 4-layer & 2-layer WP samples and Ir Black. (c) Ir-dissolution during the repeated chronoamperometry for 4-layer WP samples and Ir Black. The slope of this plot implicates the Ir-dissolution rate. Ir 4f XPS after 500 cycles stability test of (d) nanowire fabricated by S-nTP and (f) Ir black, showing the increased proportion of hydrated iridium oxide than before stability test. The SAED patterns imply no change of crystal structure in the body despite amorphized surface of the (e) P1200 nanowire (g) Ir black. The scale bars in (e) and (g) are 5 1/nm, and those in the insets of (e) and (g) are 200 nm and (b) 20 nm.

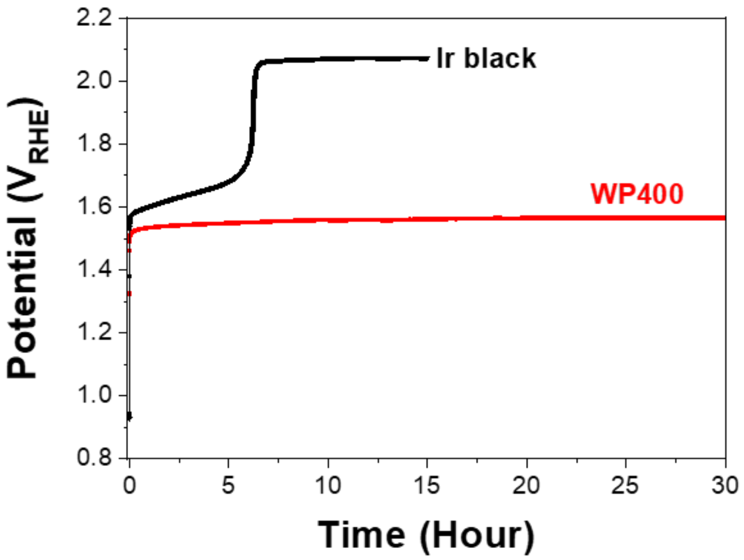

Supplementary Figure 17| Chronopotentiometry of Ir black and WP 400.

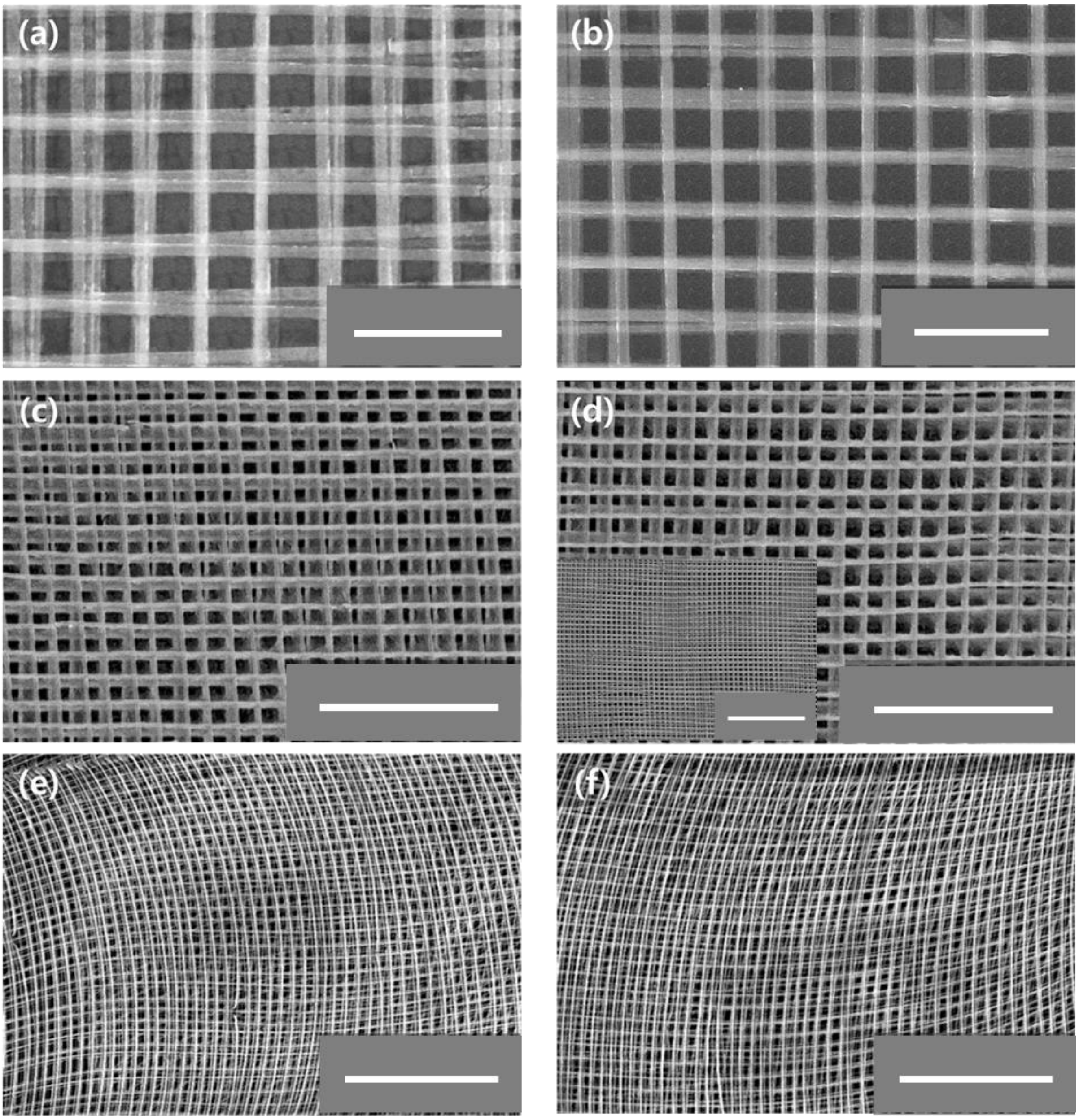

Supplementary Figure 18| Maintenance of Woodpile Nanostructure after Stability Test. SEM images of WP samples (a), (c), (e) before and (b), (d), (f) after 500 cycles of repeating chronoamperometry. The pairs (a) & (b), (c) & (d), and (e) & (f) correspond to the WP1200, WP P400, and WP P200 samples, respectively. The maintenance of the woodpile nanostructure can be confirmed with SEM images taken from the samples on the glassy carbon electrode, which can be separated from the Teflon pole of the rotating disk electrode. All of the scale bars indicate 3 μm except the 6 μm scale bar in the inset of (d).

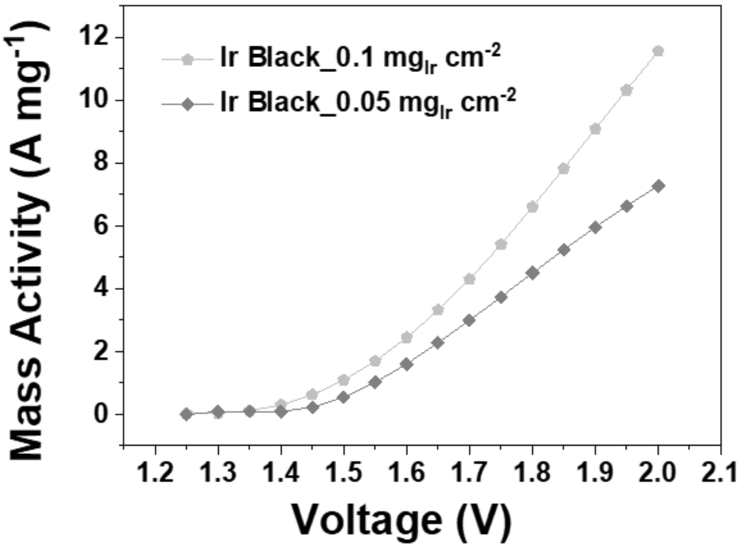

Supplementary Figure 19| Mass activities of Ir Black nanoparticles in PEMWE. With a loading amount of less than 0.05 mg cm^{-2}, Ir black nanoparticles were rarely sprayed uniformly on the substrate for the PEMWE analysis despite deliberate control of the ratio among components in the slurry. This factor might have led to the loss in performance observed when reducing the anode loading by 50%, from 0.1 to 0.05 mg cm^{-2}, which results in a contradictory trend versus previous research[9] showing improvement in Ir utilization depending on the reduction of the anode loading by 75%, from 0.4 to 0.1 mg cm^{-2}.

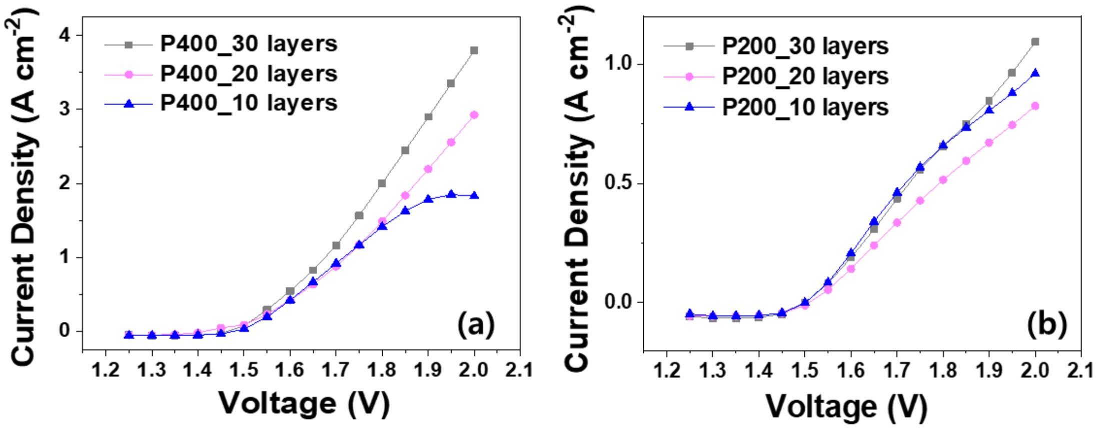

Supplementary Figure 20| I-V Curves from Single Cell Measurement. Comparison of PEMWE performance having a woodpile structured Ir thin film as an anodic catalyst. I-V curve of (a) P400 and (b) P200 showing increasing current density with increasing loading amount of Ir.

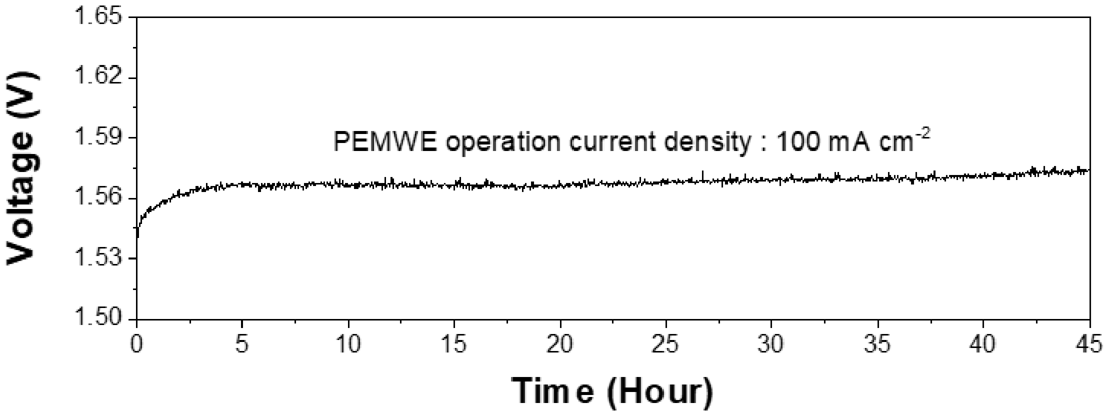

Supplementary Figure 21| Durability of PEMWE Loading WP Structures as Anode Catalyst. Stability test at 100 mA cm^{-2} in PEMWE cell for 30-layer WP1200 that generated the highest current density among our samples at the I-V curve.

Supplementary References

1 Kötz, R., Neff, H. & Stucki, S. Anodic Iridium Oxide Films XPS-Studies of Oxidation State Changes and. *J. Electrochem. Soc.* **131**, 72-77 (1984).

2 Smith, R. D., Sporinova, B., Fagan, R. D., Trudel, S. & Berlinguette, C. P. Facile photochemical preparation of amorphous iridium oxide films for water oxidation catalysis. *Chem. Mater.* **26**, 1654-1659 (2014).

3 Wertheim, G. K. & Guggenheim, H. J. Conduction-electron screening in metallic oxides: IrO_2. *Phys. Rev. B* **22**, 4680 (1980).

4 Sardar, K. *et al.* Water-Splitting Electrocatalysis in Acid Conditions Using Ruthenate-Iridate Pyrochlores. *Angew. Chem. Int. Ed.* **53**, 10960-10964 (2014).

5 Minguzzi, A. *et al.* Easy accommodation of different oxidation states in iridium oxide nanoparticles with different hydration degree as water oxidation electrocatalysts. *ACS Catal.* **5**, 5104-5115 (2015).

6 Achkar, A. *et al.* Bulk sensitive x-ray absorption spectroscopy free of self-absorption effects. *Phys. Rev. B* **83**, 081106 (2011).

7 Aziz, H. & Tafreshi, H. V. Competing forces on a liquid bridge between parallel and orthogonal dissimilar fibers. *Soft Matter* **15**, 6967-6977 (2019).

8 Soleimani, M., Hill, R. J. & van de Ven, T. G. M. Capillary Force between Flexible Filaments. *Langmuir* **31**, 8328-8334 (2015).

9 Alia, S. M. *et al.* Activity and durability of iridium nanoparticles in the oxygen evolution reaction. *J. Electrochem. Soc.* **163**, F3105-F3112 (2016).
